# Deep learning for gastroscopic images: computer-aided techniques for clinicians

**DOI:** 10.1186/s12938-022-00979-8

**Published:** 2022-02-11

**Authors:** Ziyi Jin, Tianyuan Gan, Peng Wang, Zuoming Fu, Chongan Zhang, Qinglai Yan, Xueyong Zheng, Xiao Liang, Xuesong Ye

**Affiliations:** 1 Biosensor National Special Laboratory, Key Laboratory of Biomedical Engineering of Ministry of Education, College of Biomedical Engineering and Instrument Science, Zhejiang University, Hangzhou, 310027 People’s Republic of China; 2 Hangzhou Center for Medical Device Quality Supervision and Testing, CFDA, Hangzhou, 310000 People’s Republic of China; 3 Department of General Surgery, Sir Run-Run Shaw Hospital, School of Medicine, Zhejiang University, Hangzhou, 310016 People’s Republic of China

**Keywords:** Deep learning, Gastroscopy, Stomach, Computer-aided

## Abstract

Gastric disease is a major health problem worldwide. Gastroscopy is the main method and the gold standard used to screen and diagnose many gastric diseases. However, several factors, such as the experience and fatigue of endoscopists, limit its performance. With recent advancements in deep learning, an increasing number of studies have used this technology to provide on-site assistance during real-time gastroscopy. This review summarizes the latest publications on deep learning applications in overcoming disease-related and nondisease-related gastroscopy challenges. The former aims to help endoscopists find lesions and characterize them when they appear in the view shed of the gastroscope. The purpose of the latter is to avoid missing lesions due to poor-quality frames, incomplete inspection coverage of gastroscopy, etc., thus improving the quality of gastroscopy. This study aims to provide technical guidance and a comprehensive perspective for physicians to understand deep learning technology in gastroscopy. Some key issues to be handled before the clinical application of deep learning technology and the future direction of disease-related and nondisease-related applications of deep learning to gastroscopy are discussed herein.

## Introduction

Gastric disease is a major health problem, with gastric cancer ranking second among the leading causes of cancer-related deaths [1]. Gastroscopy is the main technical method used to diagnose and screen many gastric diseases, and is the gold standard. Gastroscopy uses a thin, soft tube to extend into the stomach, enabling endoscopists to directly observe stomach lesions. It reflects the condition of the examined part and can confirm a diagnosis through pathological biopsies of suspicious lesions. It is the preferred method for examining gastric lesions.

However, endoscopists may make incorrect observations during gastroscopy due to fatigue caused by long working hours or inexperience. Several imaging modalities, such as narrow-band imaging (NBI), magnifying endoscopy (ME), autofluorescence imaging (AFI), and 3D imaging, have emerged. While these new technologies have improved the diagnostic capabilities of gastroscopy, endoscopists should be trained on how to effectively use them.

Therefore, a computer-aided diagnosis system has been developed to improve gastroscopy efficiency and quality in daily clinical practice, becoming a “third eye” for endoscopists. In recent years, deep learning technology has significantly improved the performance of computer-aided diagnosis systems due to continuous breakthroughs in algorithms, hardware performance, computing power, and the accumulation of several labelled endoscopic image datasets.

**Fig. 1:**
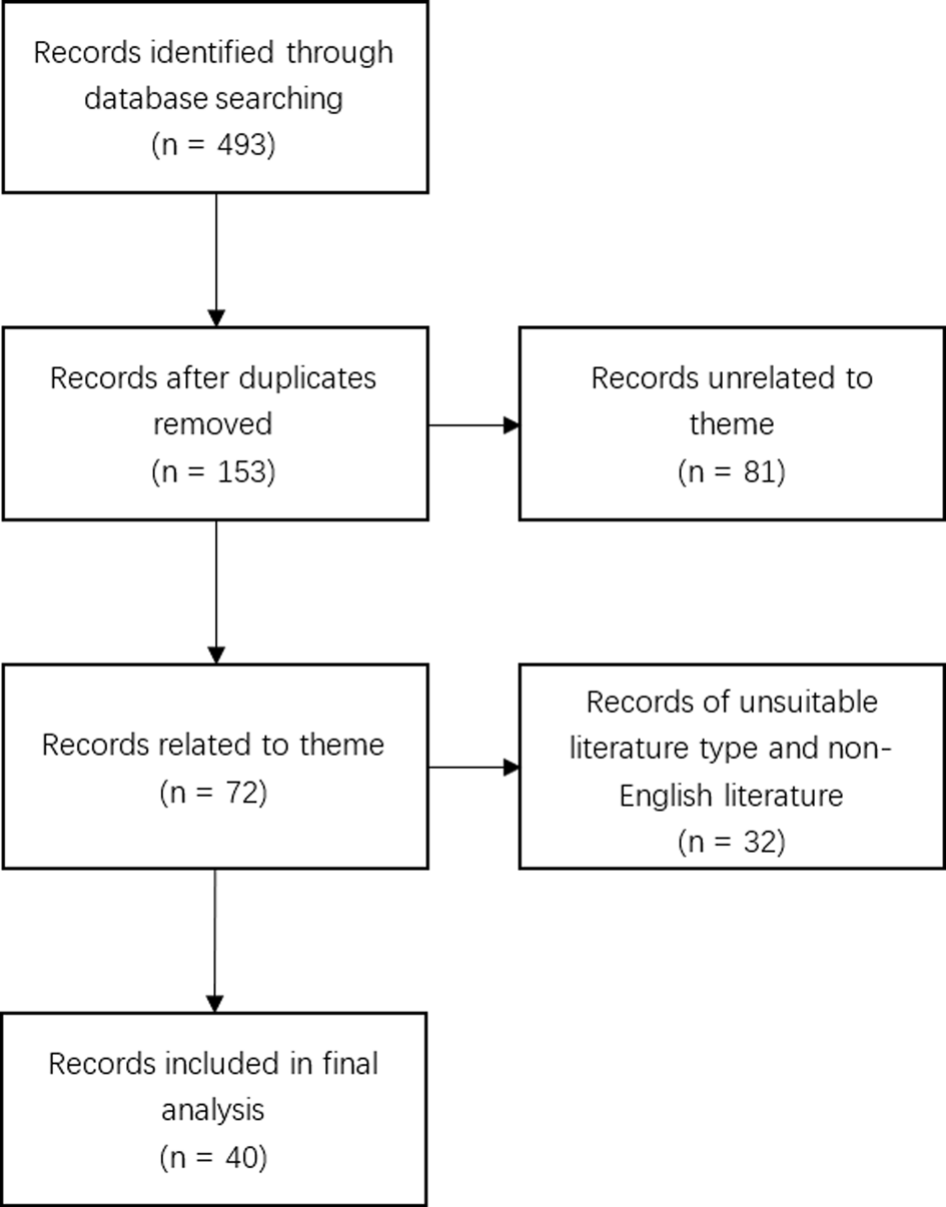
Diagram of the screening process of publications included in the analysis of this review. Duplication means that the same record is retrieved using different keywords. Relation means that the record applies deep learning technology in gastroscopy image processing (excluding wireless capsule endoscopy)

This review included relevant works published between 2018 and 2020 from the PubMed and Web of Science databases. The keywords “endoscopy gastric artificial intelligence”, “endoscopy gastric computer vision”, “endoscopy gastric convolutional neural network”, “endoscopy gastric deep learning”, “endoscopy stomach artificial intelligence”, “endoscopy stomach computer vision”, “endoscopy stomach convolutional neural network” and “endoscopy stomach deep learning” were used. A total of 493 publications were identified from the database search, and 40 manuscripts were included in the final analysis after screening (as shown in Fig. 1). This review summarizes the on-site application of deep learning during gastroscopy in recent years to provide technical guidance and a comprehensive perspective for physicians to understand what deep learning (DL) technology can do and how that role is achieved.

Some technical concepts, common networks, and algorithms used in developing a gastroscopy-assisted system are introduced in Chapter II. Details of the four main tasks of gastric image analysis using deep learning technology is presented, respectively. Chapter III summarizes existing deep learning applications for solving disease-related challenges in gastroscopy. With these technologies, endoscopists can identify, locate and diagnose lesions that appear in the viewshed of gastroscopy more accurately. Specifically, gastric diseases are classified into Helicobacter pylori, gastric cancer and other precancerous conditions, which are stated in “Helicobacter pylori”, “Gastric cancer” and “Precancerous conditions” sections, respectively. Then, Chapter IV presents the deep learning applications not directly related to diseases. They help endoscopists screen keyframes from the gastroscopic video stream and comprehensively inspect the entire surface of the oesophagus and stomach. These DL models prevent endoscopists from ignoring lesions that do not appear in the viewshed of the gastroscope or misdiagnosing lesions in poor-quality frames. “Informatic frame screening”, “Anatomical classification”, “Artefact detection” and “Depth estimation and 3D reconstruction of the stomach” sections introduced the application of deep learning for informatic frame screening, anatomical classification, artefact detection and depth estimation in gastroscopy, respectively. Chapter V shows the analysed current publications in the research field and indicates the key issues to be addressed before the clinical application of the technology. Furthermore, future perspectives for DL application in disease-related and nondisease-related gastroscopy as well as promising DL technologies and approaches are proposed. Finally, the development trend of DL-based assisted systems in real-time gastroscopy to provide on-site support is discussed.

## Technical aspects of deep learning in gastroscopy

Deep learning is a state-of-the-art (SOTA) machine learning technique. Before deep learning, machine learning mainly used handcrafted features, where image patterns such as colour and texture were encoded in a mathematical description. A classifier was then used to analyse the features of each image category during a training process and to classify a new input image. A DL architecture has several hidden layers and can automatically extract and identify numerous high-level, complex features that a traditional machine learning (ML) method cannot analyse.

**Fig. 2:**
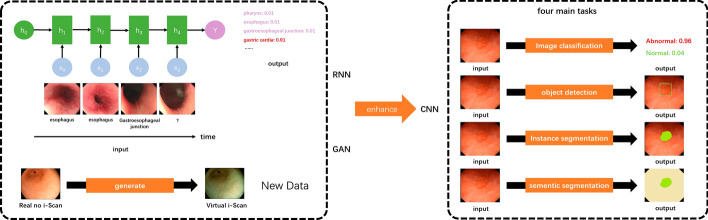
Illustration of the four main tasks of gastric image analysis using deep learning technology. A CNN is the most popular neural network that has already been applied to gastroscopic image analysis. Recently, RNNs and GANs have been used to enhance the performance of CNN-based gastroscopic image processing methods for the four main tasks

Convolutional neural networks (CNNs) are the first and most commonly used deep neural networks for gastric image analysis. A CNN has a unique effect on image processing. Its structure includes convolutional layers, pooling layers, and fully connected layers. CNN applications for gastric image analysis can be grouped into four main tasks based on the challenges endoscopists encounter in clinical practice: image classification, object detection, semantic segmentation, and instance segmentation. Figure 2 illustrates the difference among the four main tasks. Recently, recurrent neural networks (RNNs) and generative adversarial networks (GANs) have also been used to further improve the performance of CNN-based gastroscopic image processing methods with regard to these clinical challenges. Unlike CNNs, RNNs efficiently process time-series data because they can remember historical information. By combining the information from several adjacent oesophagogastroduodenoscopy (EGD) video frames, focusing on the time sequence of the input and the connection between the previous and next frames, a better effect is achieved in gastroscopic image analysis [2]. The internal memory structure of an RNN meets such a scenario. Gated recurrent unit networks (GRUs) [3] and long short-term memory (LSTM) networks [4] are commonly used RNN architectures based on practical performance. Generative adversarial networks (GANs) introduce the confrontation idea in deep learning. The discriminant model and the generative model are the two confrontation sides. The discriminant model accurately distinguishes real data from generated data, and the generative model generates new data that conform to the probability distribution of real data. A GAN can effectively generate new data similar to real data via the adversarial training of the two neural networks. The function of GANs in gastroscopic image analysis mainly includes image data enhancement [5], image style transfer [6], and image restoration [7] due to the inadequate endoscopic data and poor-quality frames in EGD videos. Typical GAN algorithms include DCGANs [8], CGAN [9], and CycleGAN [10]. [11] lists the famous GANs. “Image classification task”, “Object detection task”, “Semantic segmentation task”, and “Instance segmentation task” section provide a detailed introduction to the four main tasks of gastric image analysis using deep learning technology.

### Image classification task

An image classification task determines the category of a given input image at the image level. It is a basic task in high-level image understanding and can be divided into binary- and multi-classification tasks. After multiple convolution-and-pooling operations via a CNN, an image is classified in the output layer following the requirements. The activation function of the output layer is the only difference between binary- and multi-classification tasks. An image classification task for gastric image analysis mainly includes determining whether a frame is an analysable information frame [12, 13] or contains a lesion [14–22], determining frame anatomical position [2, 12, 18, 23–25], and the classification of lesion features [19, 26–39]. Some classification networks with high performance in natural image classification, including AlexNet [40], VGG [41], GoogLeNet (Inception) series [42–45], ResNet [46], ResNeXt [47], DenseNet [48], SENet [49], SqueezeNet [50], and EfficientNet [51], can be used in EGD image classification.

### Object detection task

Object detection detects all objects in an image, giving their location information using a bounding box and classifying each object. An object detection network uses a classification network with a powerful feature extraction capability as its backbone. It achieves its goals by changing the output layer structure. An object detection task for gastroscopic images involves detecting, boxing, and classifying lesions [52–55] and artefacts [7, 56], and the anatomical structure of the stomach [13]. Two-stage algorithms using candidate regions such as RCNN [57], SPP-Net [58], fast RCNN [59], and faster RCNN [60] and one-stage algorithms based on regression such as YOLO series [61–65], SSD [66], CornerNet [67], ExtremeNet [68] and CenterNet [69] are the two main object detection algorithms. While some classic object detection networks have achieved good results in gastroscopic image analysis, some SOTA algorithms, such as EfficientDet [70] and CentripetalNet [71], with higher performance and less calculation time, should be considered because a DL model will finally be used for clinical real-time videos.

### Semantic segmentation task

Semantic segmentation is a more fine-grained task than object detection that determines each pixel class of an entire image. It classifies an image pixel-by-pixel. The height and width of the output are the same as those of the input image. The number of channels equals the number of categories, representing each spatial location category (pixel-by-pixel classification). It mainly segments a lesion [35, 39, 72, 73] and the artefact [56] boundary and estimates the depth of endoscopic images and 3D reconstruction of the stomach [74] in gastroscopic image analysis. Several classic algorithms, such as FCNs [75], SegNet [76], U-Net [77], and DeepLeb series [78–81], have been used in this field.

### Instance segmentation task

Instance segmentation distinguishes different instances from the same category. For instance, semantic segmentation only predicts the pixels of multiple lesions as a category of "lesions", but instance segmentation distinguishes each pixel from multiple lesions such as “lesion 1”, “lesion 2” and “lesion 3”. Instance boxing using an object detection algorithm and semantic segmentation on each bounding box is used to realize instance segmentation. An instance segmentation task mainly detects the lesions and delineates their margin [82]. Mask RCNN [83], PANet [84], and CentripetalNet [71] are the superior algorithms for this task.

## Deep learning application to disease-related gastroscopy challenges

At this time, available DL models are not like human endoscopists, who can screen multiple diseases and take a biopsy for qualitative analysis at the same time during a gastroscopy. Most gastroscopy DL applications focus on a single disease and achieve a specific clinical task. Therefore, we divide stomach diseases into three categories, Helicobacter pylori (HP), gastric cancer (GC), and other precancerous diseases, and introduce the application of DL in solving specific clinical tasks related to each.

### Helicobacter pylori

**Fig. 3:**
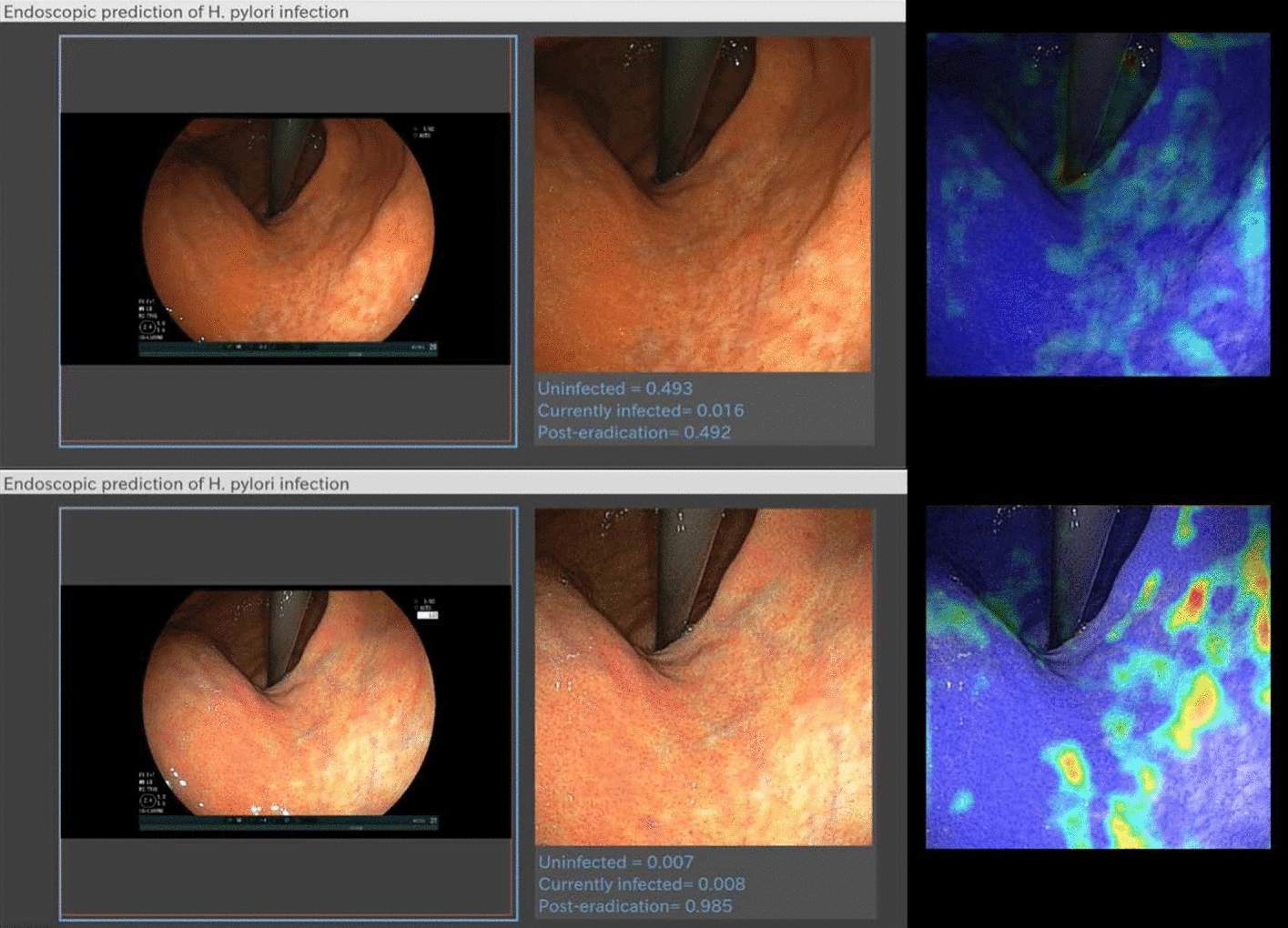
Diagnosis with a CAD system for the endoscopic video of a post-eradication subject. The computer-aided diagnosis system for white-light imaging (WLI-CAD, upper side) returned a prediction value of 0.492 for a post-eradication status, which turned out to be an incorrect prediction. However, linked colour imaging (LCI-CAD, lower side) returned a prediction value of 0.985 for a post-eradication status, which turned out to be the correct prediction. The lower heatmap demonstrates that hot spots were drawn against the contrast between a pale reddish tone and a whitish tone of the gastric mucosa in the captured LCI image. (Reproduced with permission from Ref. [37]. Copyright 2020 Springer Nature Publishing)

Helicobacter pylori infection causes chronic atrophic gastritis and intestinal metaplasia, which increase the risk of gastric cancer. Approximately 90% of noncardia gastric cancers are related to HP infection [85–88]. The redness and swelling of the gastric mucosa during an endoscopy inspection can be used to diagnose an HP infection. However, it is time-consuming, and the accuracy of the results depends on the skill of the endoscopist. Recently, some articles reported a method that detects and diagnoses HP infection using a deep learning model. Itoh et al. [14] first developed a convolutional neural network using GoogLeNet to differentiate HP-positive from HP-negative in white-light imaging (WLI) images and showed a sensitivity, specificity, and area under the curve (AUC) of 86.7%, 86.7%, and 0.956, respectively. In addition, Zheng et al. [20] developed a CNN model using ResNet-50 to evaluate HP infection and obtained similar results. Shichijo et al. [28] constructed a convolutional neural network (GoogLeNet) to ascertain HP infection statuses, including HP-positive, HP-negative, and HP-eradicated. A total of 23,699 images from 847 patients were used to validate the algorithm and showed a diagnostic accuracy of 80%, 84%, and 48% for negative, eradicated, and positive, respectively, similar to the results of experienced endoscopists [89]. Nakashima et al. [15] constructed a GoogLeNet-based DL model to predict the HP infection status in WLI, blue-light imaging (BLI), and linked colour imaging (LCI) images. The AUCs were 0.66, 0.96, and 0.95 for WLI, BLI, and LCI, respectively. Nakashima et al. [37] developed two DL models using a 22-layer skip-connection architecture to classify the HP infection status into three similar categories for WLI and LCI images (as shown in Fig. 3). A validation dataset of endoscopic videos of 120 subjects was developed to evaluate computer-aided diagnosis (CAD) systems. Comparisons revealed that LCl-based DL diagnoses were more accurate than WLI-based DL diagnoses [uninfected (84.2% vs. 75.0%), currently infected (82.5% vs. 77.5%) and post-eradication (79.2% vs. 74.2%)], indicating that a DL model with image-enhanced endoscopy is a more powerful image diagnostic tool for HP infection than conventional white-light endoscopy.

### Gastric cancer

Gastric cancer is a common gastrointestinal tumour with rapid progress and high modality that seriously threatens human life and health [90, 91]. Gastroscopy and pathological biopsy are the gold standards for gastric cancer diagnosis. However, gastroscopy depends on equipment and the diagnostic ability of endoscopists. Therefore, several deep learning models have been recently developed to assist in diagnosing various aspects of gastric cancer.

#### GC detection

**Fig. 4:**
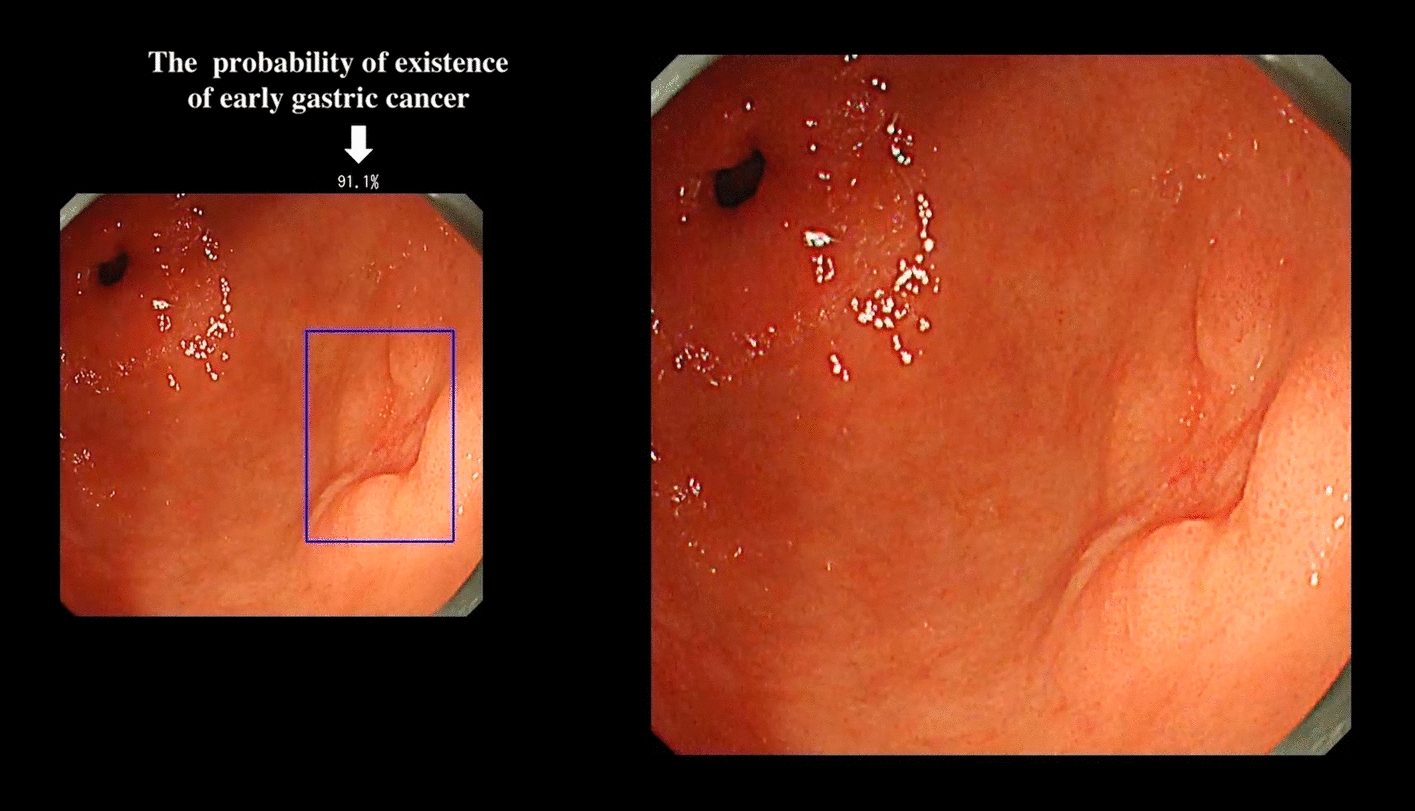
Sample images for the early detection of gastric cancer using a convolutional neural network (CNN) system. Real‐time detection using the CNN system is displayed on the left‐hand‐side screen (video image). (Reproduced with permission from Ref. [53]. Copyright 2018 John Wiley and Sons Publishing)

Gastric cancer prognosis is related to detection time. The 5-year survival rate of advanced gastric cancer is less than 30%, even after surgical treatment [92]. Meanwhile, radical treatment under endoscopy can be used for most early gastric cancers with a 5-year survival rate of more than 90% [93]. However, early gastric cancer usually does not have obvious characteristics under endoscopy; only slight local regional mucosal changes occur, which are difficult to detect. Hirasawa et al. [52] first developed a CNN using single-shot multibox detection (SSD) to automatically detect gastric cancer in endoscopic images. A total of 13,584 endoscopic images were used, and the model could correctly detect 71 of 77 GC lesions (92.2% sensitivity) in 2296 stomach images requiring only 47 s. The unidentified lesions were superficially downregulated and differentiated-type intramucosal cancers, which can be easily misdiagnosed as gastritis. Hirasawa et al. also applied the technology to real-time GC detection in videos [53]. The CNN correctly detected 64 of 68 EGC lesions (94.1% sensitivity) from 68 endoscopic submucosal dissection (ESD) procedures for EGC in 62 patients. The median time for lesion detection after the first appearance on the screen was 1 s. A sample image for the early detection of gastric cancer using their CNN system is shown in Fig. 4. Moreover, they compared the detection ability between the CNN and endoscopists [55]. An independent test set of 2940 images from 140 cases was used for validation. The CNN system showed a significantly higher sensitivity than the 67 endoscopists (58.4% vs. 31.9%) at a faster detection speed (45.5 s vs. 173.0 min). Sakai et al. [16] proposed a GoogLeNet-based model to detect EGC under WLI. The accuracy, sensitivity, and specificity were 87.6%, 80.0%, and 94.8%, respectively. Luo et al. developed another DL system named GRAIDS using DeepLabv3 +  + by [72] to detect upper gastrointestinal (GI) cancer. This multicentre, case–control study was performed in six hospitals of different tiers in China. The model was trained and tested on 1,036,496 endoscopic images from 84,424 individuals, which is the largest dataset in this research area to date. GRAIDS showed a sensitivity comparable to that of expert endoscopists (0.942 vs. 0.945) and was superior to competent (0.858) and trainee (0.722) endoscopists. Wu et al. [18] built a deep convolutional neural network (DCNN) to detect EGC in real-time unprocessed EGD videos and designed a man–machine competition. The DCNN detected EGC with an accuracy of 92.5%, a sensitivity of 94.0%, a specificity of 91.0%, a positive predictive value (PPV) of 91.3%, and a negative predictive value (NPV) of 93.8%, greater than that of endoscopists at all levels. Wang et al. constructed a cloud-based image analysis service to enhance GC screening [17]. Their study is unique due to its deployment method and result integration of all trained CNNs (AlexNet, GoogLeNet, and VGGNet) to obtain the final prediction. The sensitivity of the proposed approach (79.6%) was significantly greater than that of other single-CNN models (61.5% for AlexNet, 68.8% for GoogLeNet, and 69.7% for VGGNet). Yoon et al. [19] developed a VGG-16-based DL system to detect EGC. The model showed a sensitivity of 91.0% and an AUC of 0.981. Shibata et al. [82] developed a Mask R-CNN-based detection method for EGC. They collected 1208 healthy and 533 cancer images to perform fivefold cross-validation. The results showed 96% sensitivity with only 0.10 false positives per image, which is acceptable for endoscopists in clinical practice if the performance is not significantly influenced after being applied to video images.

#### GC diagnosis

**Fig. 5:**
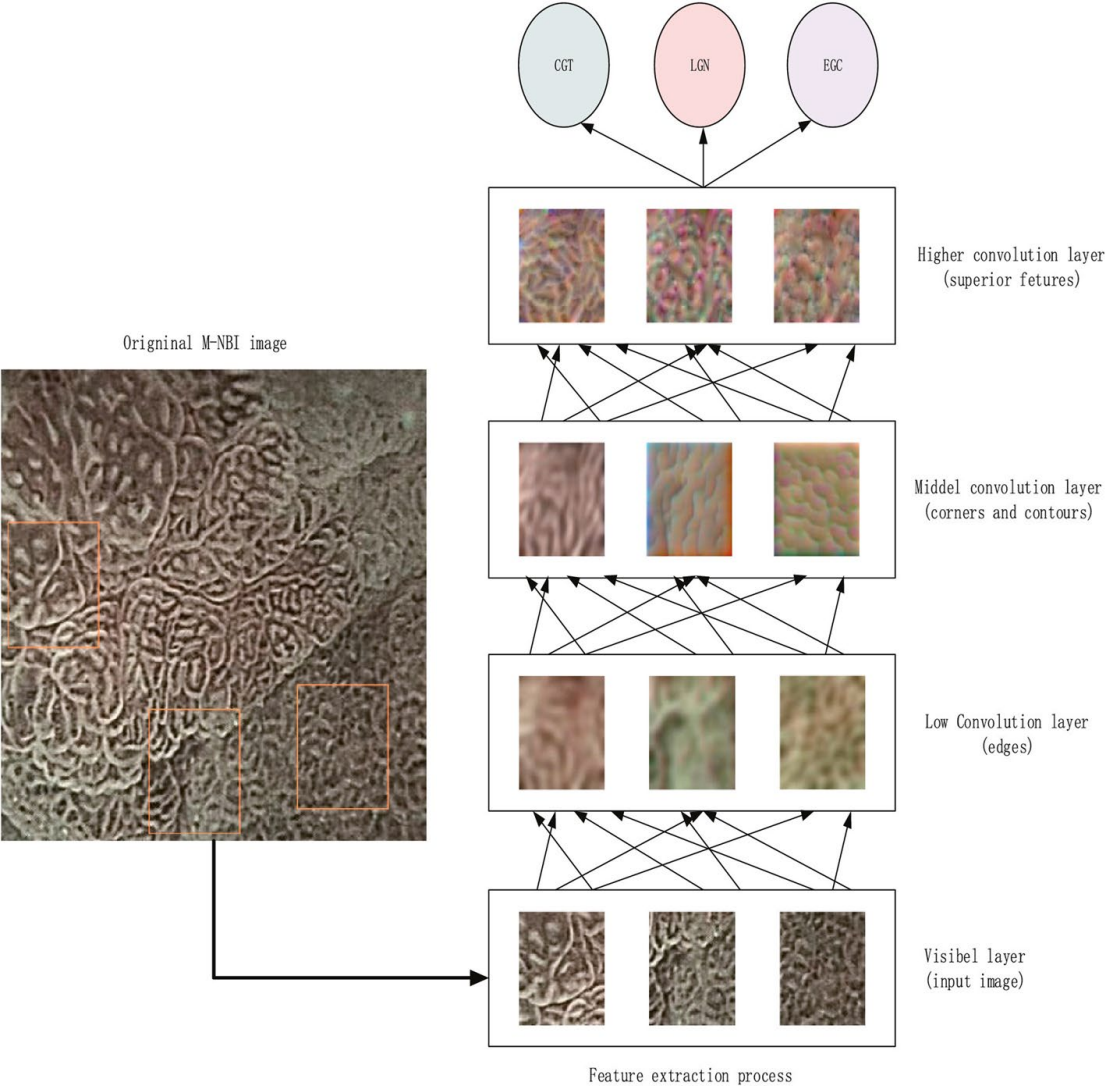
Example of an original ME-NBI image and the feature extraction procedure for its classification. The original ME-NBI image is classified into three types: CGT, LGN, and EGC. (Reproduced with permission from Ref. [36]. Copyright 2020 Elsevier Publishing)

Unlike GC detection, which emphasizes sensitivity to reduce the rate of missing lesions, in GC diagnosis, a DL model distinguishes benign lesions from GC, emphasizing accuracy, reducing unnecessary biopsies, and minimizing costs. Cho et al. [26] established CNN models using Inception-Resnet-v2 to automatically classify gastric neoplasms under WLI into five categories [advanced gastric cancer (AGC), early gastric cancer (EGC), high-grade dysplasia (HGD), low-grade dysplasia (LGD), and nonneoplasm]. The CNN model showed lower performance in the prospective validation using 200 images from 200 patients compared with the best endoscopists (five-category accuracy 76.4% vs. 87.6%; cancer 76.0% vs. 97.5%; neoplasm 73.5% vs. 96.5%) but was comparable to that of the worst endoscopist (cancer accuracy 76.0% vs. 82.0%), indicating potential clinical application in classifying gastric cancer or neoplasm. Lee et al. [27] constructed three CNNs using ResNet-50, VGG-16, and Inception-v4 to differentiate GC from gastric ulcers. ResNet-50 had the highest performance with 77.1% accuracy. Zhang et al. [39] developed a CNN system using ResNet34 and DeepLabv3 to assist the diagnosis of GC and other gastric lesions. The model was trained on 21,217 images, including five gastric conditions [peptic ulcer (PU), early gastric cancer (EGC), high-grade intraepithelial neoplasia (HGIN), advanced gastric cancer (AGC), submucosal tumours (SMTs)] and normal gastric mucosa. In addition, 1091 other images were used to evaluate the model. The diagnostic accuracy, specificity, and PPV of the CNN were higher than those of endoscopists with over 8 years of experience (accuracy: 78.7% vs. 74.2%; specificity: 91.2% vs. 86.7%; PPV: 55.4% vs. 41.7%). While GC diagnosis cannot achieve high accuracy under WLI, imaging enhancement endoscopy, such as ME-NBI, which can provide more structural information on mucosa and capillaries, is more accurate for distinguishing GC from other benign lesions, endoscopists require substantial effort to learn the skill since its efficiency relies on endoscopist experience. Therefore, a DL model in this field is extensively researched. Hu et al. [33] developed a VGG-19-based DL model (accuracy, 77.0%) to classify EGC and noncancerous lesions. Li et al. [34] also developed a CNN system using Inception-v3 to differentiate EGC from noncancerous lesions using ME-NBI images (accuracy, 90.91%; sensitivity, 91.18%; and specificity, 90.64%). Liu et al. [36] developed a ResNet-50-based CNN to classify ME-NBI endoscopic images into chronic gastritis (CGT), low-grade neoplasia (LGN), and EGC (accuracy, 0.96). Examples of the original ME-NBI image and the feature extraction procedure for its classification are provided in Fig. 5. In addition, Ueyama et al. [38] constructed a CNN using ResNet-50 to differentiate EGC from noncancerous mucosa and lesions. A total of 2300 ME-NBI images were used, and the model illustrated an extremely excellent performance. The overall accuracy, sensitivity, specificity, PPV and NPV were 98.7%, 98%, 100%, 100% and 96.8%, respectively. The total time for analysing the test dataset was only 60 s. Horiuchi et al. [31] reported a GoogLeNet-based CNN system to distinguish EGC from gastritis. A total of 1492 EGC and 1078 gastritis ME-NBI images were used for training, and 151 EGC and 107 gastritis images were used to evaluate the diagnostic ability. The accuracy of the model reached 85.3%, and the overall test speed was 0.02 s/image. Horiuchi et al. also conducted a video-based evaluation to compare the performance between expert endoscopists and the CNN model [32]. The study included 174 ME-NBI videos (87 cancerous and 87 noncancerous) and 11 experts. The CNN model achieved an accuracy of 85.1%, which was significantly higher than that of two experts, less than that of one expert, and not significantly different from that of the remaining eight experts.

#### GC type classification

**Fig. 6:**
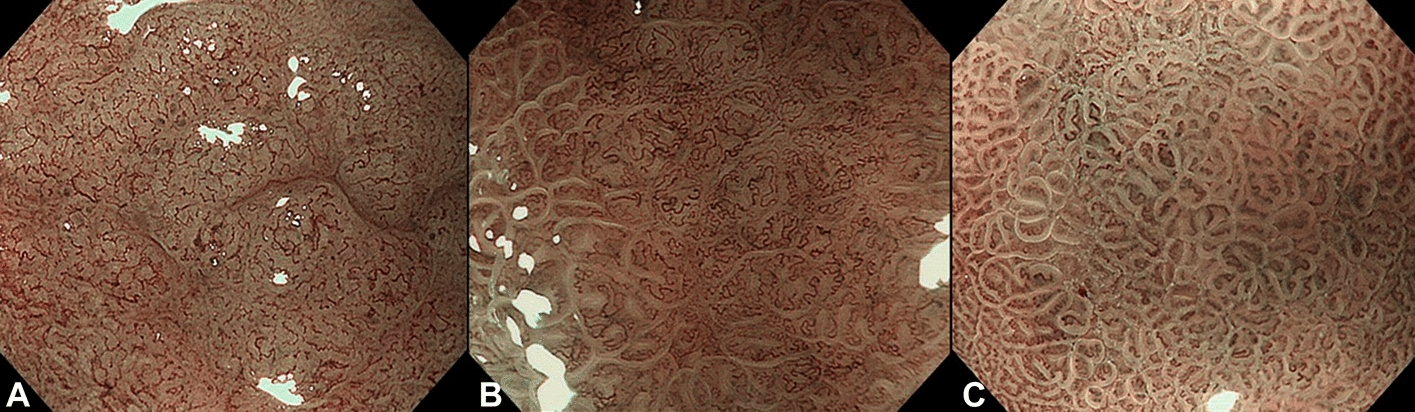
Examples of images with different differentiation statuses. **A** Cancerous image (differentiated type). **B** Cancerous image (undifferentiated type). **C** Noncancerous image. (Reproduced with permission from Ref. [32]. Copyright 2020 Elsevier Publishing)

Identifying the type of GC, such as the differentiation status, accurately is critical for determining the surgical strategy and treatment plan. GC with different differentiation statuses shows an obvious difference in images under narrow-band imaging (Fig. 6). Therefore, it can be classified using a deep learning method. Ling et al. [35] developed a real-time system using VGG-16 to accurately identify the EGC differentiation status from ME-NBI endoscopy. A total of 2217 images from 145 EGC patients and 1870 images from 139 EGC patients were retrospectively collected to train and test the CNN. The performance of the CNN was then compared with that of experts using 882 images from 58 EGC patients. The system correctly predicted the differentiation status of EGCs with an accuracy of 83.3% on the test dataset and achieved superior performance compared with the five experts (86.2% vs. 69.7%). Furthermore, the system was successfully used on real EGC videos.

#### Determination of GC invasion depth

GC invasion depth is essential in determining the treatment method. For GC in the mucosa or superficial submucosa, endoscopic submucosal dissection (ESD) can be used for radical GC treatment without surgery or chemotherapy because it is minimally invasive and requires only a short hospital stay. However, there are limitations in clinical practice because endoscopists measure the exact depth based on the overall findings and personal experience. Yoon et al. [19] used a VGG-16 model to classify EGC endoscopic images as T1a (intramucosal) or T1b (submucosal). A total of 11,686 endoscopic images were used to perform fivefold cross-validation, and the AUC for depth prediction reached 0.851. However, undifferentiated-type GC showed a lower accuracy than differentiated-type GC. Zhu et al. [29] constructed a ResNet-50-based CNN to determine the invasion depth of GC in the mucosa or superficial submucosa (M/SM1) and deep submucosa (SM2). The model obtained an overall accuracy of 89.16%, specificity of 95.56%, PPV of 89.66%, and NPV of 88.97%. The accuracy and specificity were significantly higher than those of endoscopists. Furthermore, Cho et al. [30] developed a CNN based on DenseNet-161 to discriminate the mucosa-confined and submucosa-invaded GC invasion. The model showed excellent performance. The model accurately identified 6.7% of patients who underwent gastrectomy in an external test for potential ESD, preventing unnecessary operation.

#### GC margin delineation

**Fig. 7:**
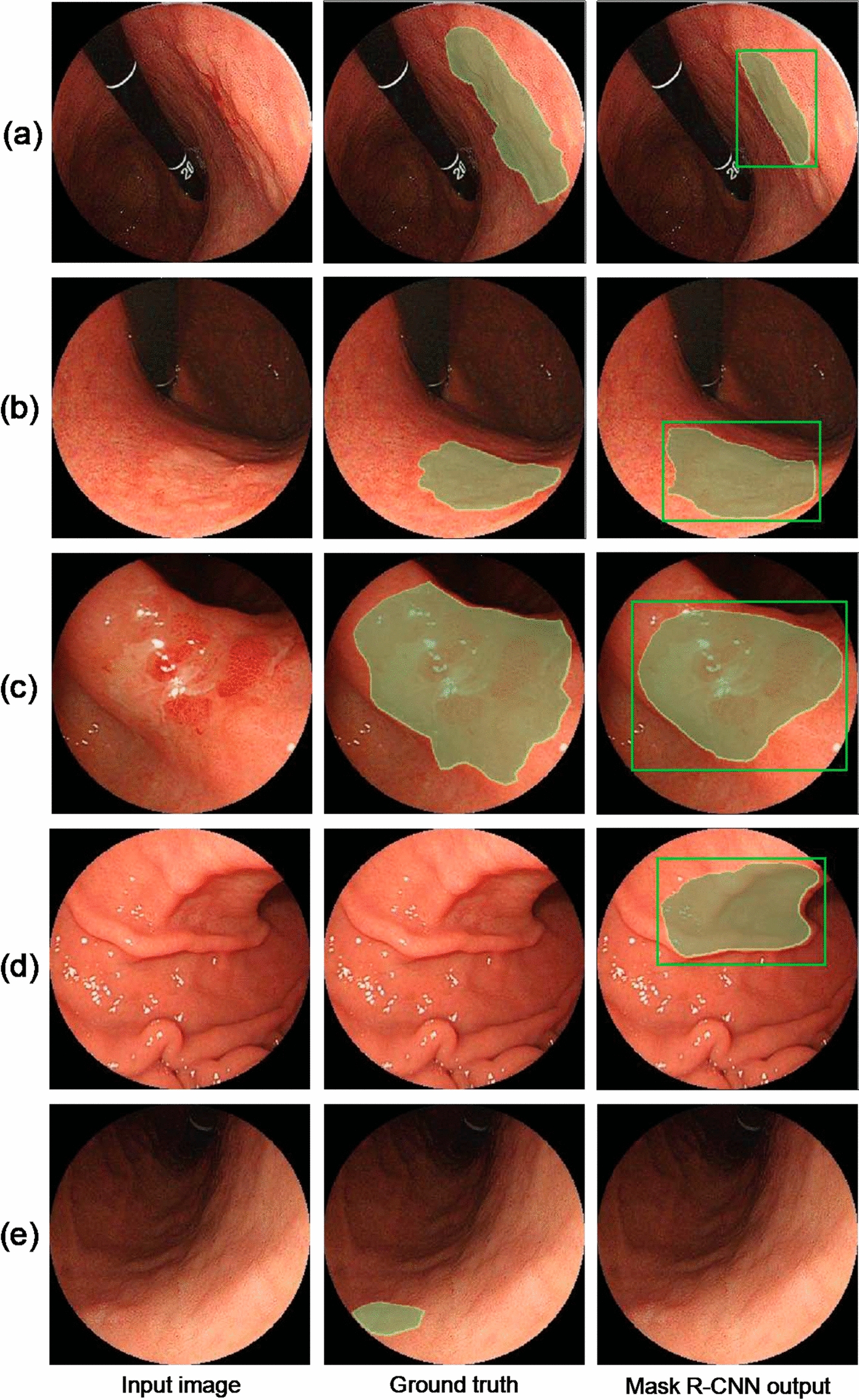
Delineation results. **a**–**c** Successful gastric cancer detection and delineation, **d** false positives (FP) in healthy subjects, and **e** false negatives in abnormal cases. (Reproduced with permission from Ref. [82]. Copyright 2020 MDPI Publishing)

It is important to first delineate the GC margin accurately before ESD to achieve endoscopic curative resection in EGC patients. An et al. [73] used a real-time fully convolutional network (UNet + +) to delineate the resection margin of EGC under indigo carmine (IC) chromoendoscopy (CE) or white-light endoscopy (WLE). The system (ENDOANGEL) showed an accuracy of 85.7% on the CE images and 88.9% on the WLE images under an overlap ratio threshold of 0.60 relative to expert-labelled manual markers. The system was also tested on ESD videos, and ENDOANGEL predicted the regions covering all areas of high-grade intraepithelial neoplasia and cancers. An et al. also developed a real-time system to accurately delineate EGC margins on ME-NBI endoscopy using the same UNet +  + architecture [35]. A total of 928 images from 132 EGC patients and 742 images from 87 EGC patients were used to train and test the system. The model showed an accuracy of 82.7% in differentiated EGC and 88.1% in undifferentiated EGC under an overlap ratio of 0.80. This system achieved superior performance compared with experts and was successfully tested on real-time EGC videos. Shibata et al. [82] developed a segmentation method using Mask R-CNN for EGC regions (as shown in Fig. 7). A total of 1208 healthy and 533 cancer images were collected, and the performance was evaluated via fivefold cross-validation. The average Dice index was 71%, indicating that the proposed scheme is useful for evaluating the invasion region.

### Precancerous conditions

**Fig. 8:**
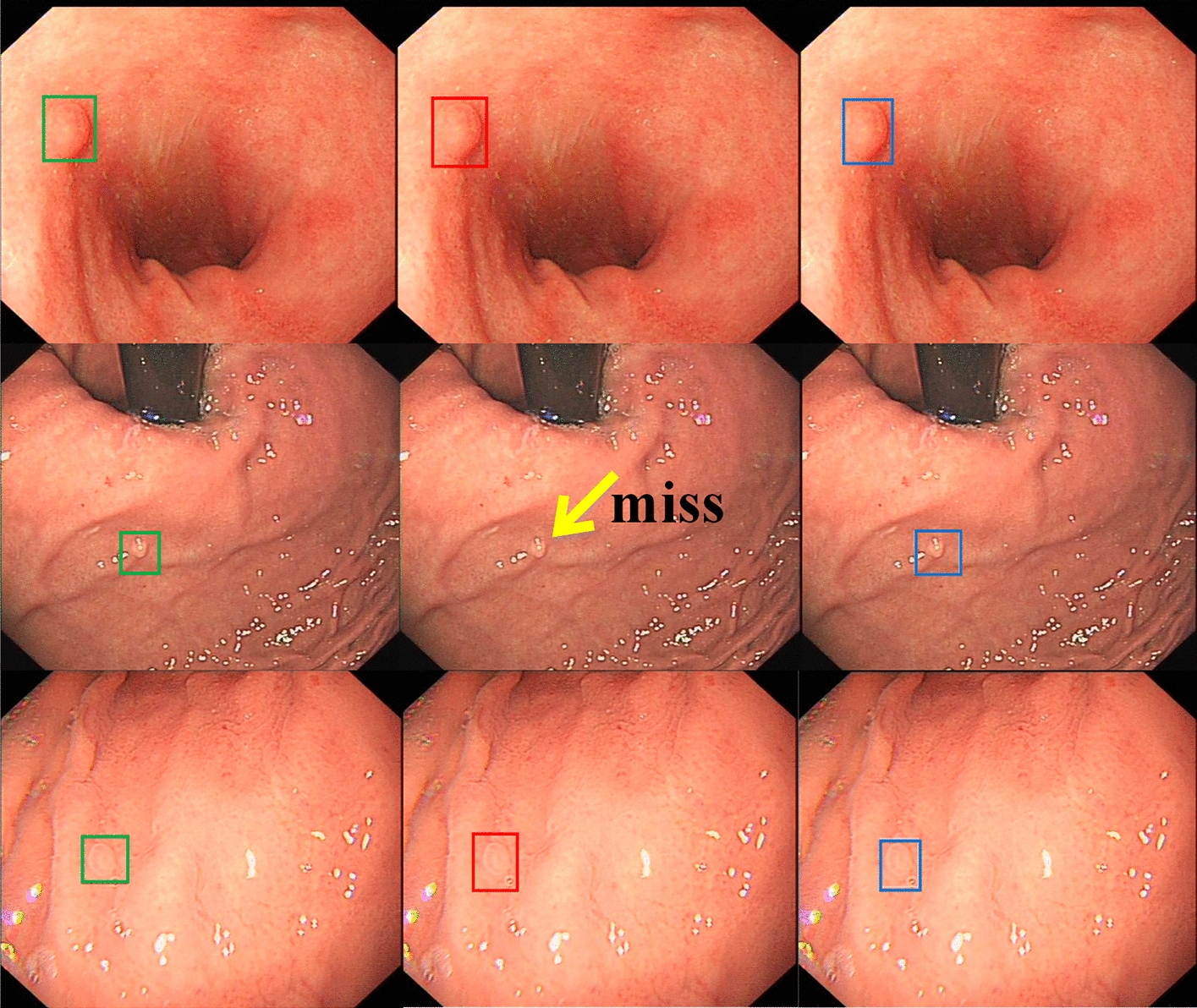
Some examples of detection results using a conventional SSD and SSD-GPNet. The first column shows the "ground truth" as labelled by experienced doctors. The second column shows the SSD detection results. The last column shows the SSD-GPNet detection results (Reproduced with permission from Ref. [54]. Copyright 2019 Zhang et al.)

**Fig. 9:**
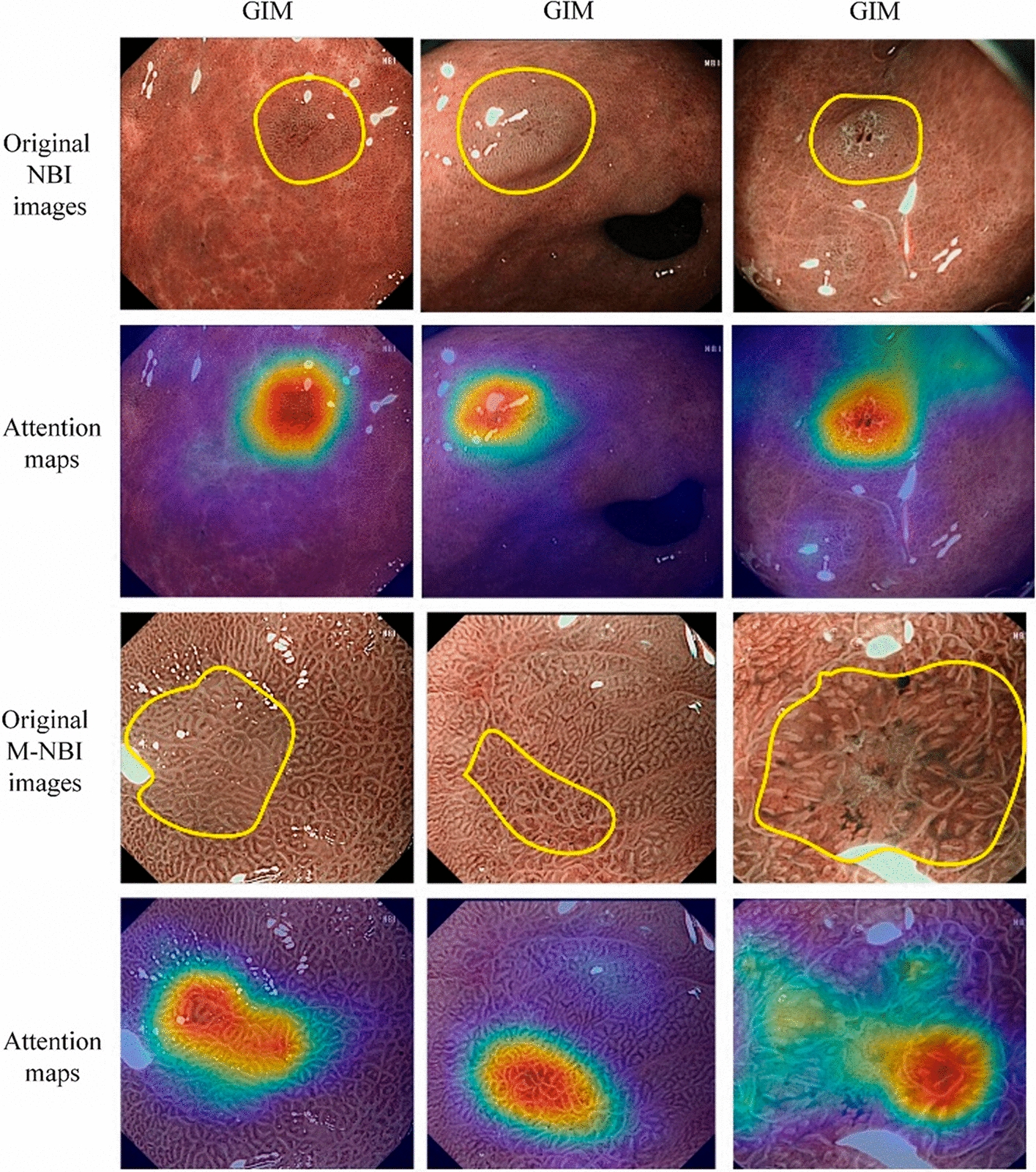
Attention maps generated using the Grad-CAM method. The yellow wireframe areas are the lesion regions of GIM annotated by an experienced endoscopist. The attention map is a heatmap laid over the original image, where a warmer colour indicates a higher contribution to the classification decision. (Reproduced with permission from Ref. [22]. Copyright 2020 Elsevier Publishing)

**Fig. 10:**
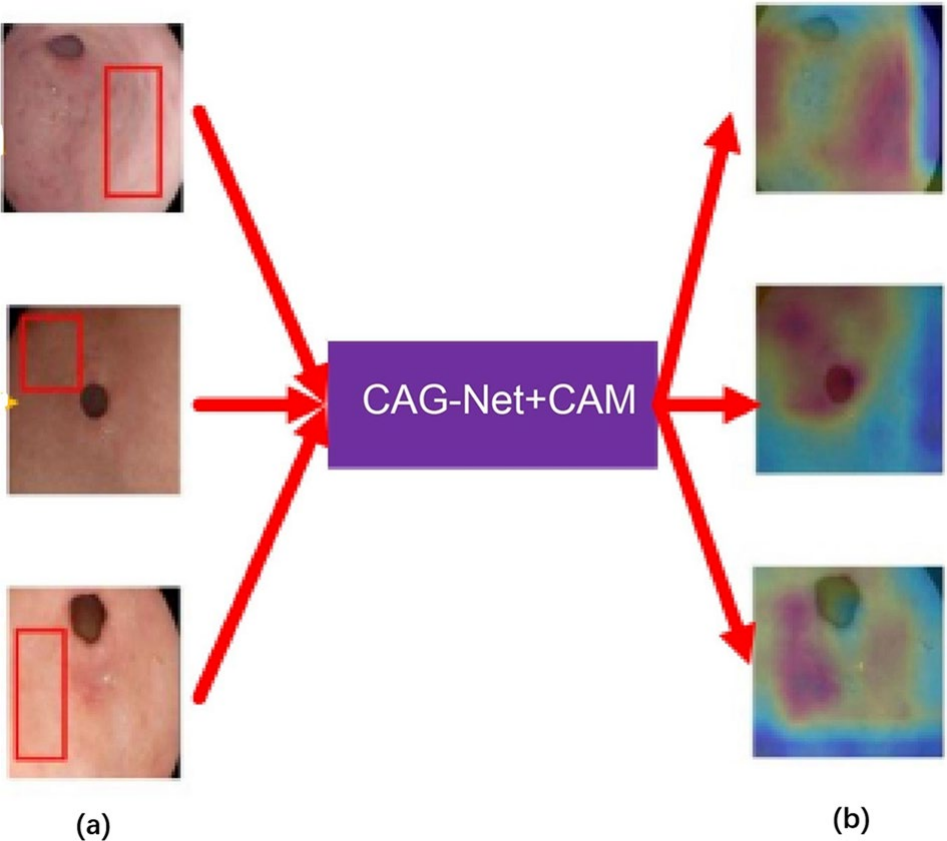
Interpretable thermodynamic maps for the automatic diagnosis of chronic atrophic gastritis. **a** Original images. The red boxes are the areas of focus labelled by a doctor. **b** Heatmaps generated with class activation mapping. The orange–red regions of the heatmaps are consistent with the atrophic mucosa labelled by the doctors according to pathological results (Adapted with permission from Ref. [21] Copyright 2020 Elsevier Publishing)

While most precancerous conditions in the stomach are benign and harmless, they can develop into gastric cancer if not diagnosed and treated early. Zhang et al. [54] developed an SSD-based CNN named SSD-GPNet to detect gastric polyps. The network could realize real-time polyp detection with 50 fps and improve the mean average precision (mAP) to 90.4%. Some examples of the results are shown in Fig. 8. Further experiments showed that their network has an excellent performance in improving polyp detection by over 10%, especially for small polyps. Yan et al. [22] constructed a CNN (EfficientNetB4) using NBI and ME-NBI images to diagnose gastric intestinal metaplasia (GIM). A separate dataset of 477 images (242 GIM and 235 non-GIM) was used as the test set. The performance of the system was not significantly different from that of human experts (sensitivity 91.9% vs. 86.5%; specificity 86.0% vs. 81.4%; accuracy 88.8% vs. 83.8%). Figure 9 displays the classification decision procedure of a CNN using the Grad-CAM [94] method. Zhang et al. [21] constructed a CNN named CAG-Net using DenseNet121 to improve the diagnostic rate of chronic atrophic gastritis. Fivefold cross-validation was used to train and verify the model (3042 atrophic gastritis images and 2428 normal images). The diagnostic accuracy, sensitivity, and specificity of the model were 0.942, 0.945, and 0.940, respectively. The detection rates of mild, moderate, and severe atrophic gastritis were 93%, 95%, and 99%, respectively. Figure 10 shows interpretable thermodynamic maps of the CAG automatic diagnosis procedure.

## Deep learning application to nondisease-related gastroscopy challenges

The DL technologies discussed in Chapter III can reach or even exceed experienced endoscopists in many disease-related clinical tasks. However, if a lesion has never entered the viewshed of the gastroscope due to incomplete inspection or the poor quality of video frames during gastroscopy, these systems do not work at all. Therefore, some deep learning technologies not directly related to gastric diseases have also been applied to improve the quality of gastroscopy.

### Informatic frame screening

**Fig. 11:**
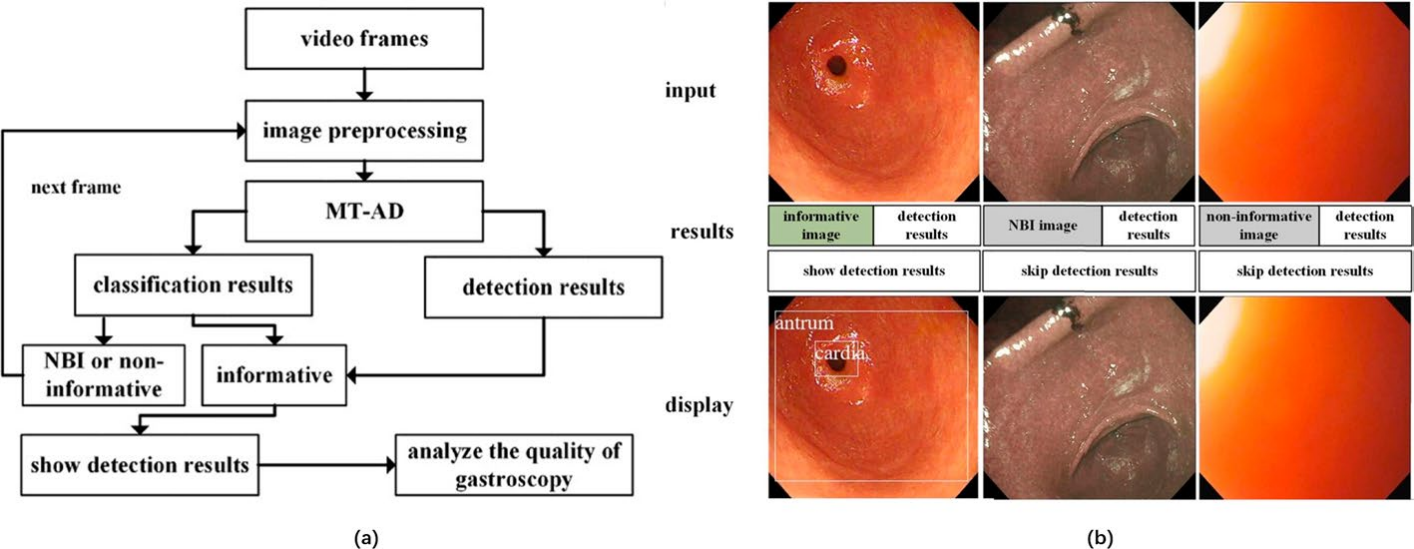
Upper gastrointestinal anatomy detection with multitask convolutional neural networks. **a** Workflow of the proposed method. **b** Examples of MT-AD detection results (Adapted with permission from Ref. [13] Copyright 2019 John Wiley and Sons Publishing)

The video stream in clinical endoscopy can output 30 or 60 image frames per second, including many useless frames with no information. A deep learning model cannot analyse useless frames because of poor image quality or inappropriate imaging modalities. The useless frames show uncredible results, mislead endoscopists, waste considerable computing power, and decrease the real-time performance of the system. Wu et al. [12] developed a DCNN using VGG-16 to identify informatic frames. A total of 12,220 in vitro, 25,222 in vivo*,* and 16,760 unqualified EGD images from over 3000 patients were used for training the network to identify whether a frame was outside the body with high quality for the next-step analysis. A total of 3000 images (1000 per category) were randomly selected to test the model (accuracy, 97.55%). In addition, Zhang et al. [13] constructed a model of seven convolutional layers, one max-pooling layer, and one fully connected layer to classify video frames into three categories (NBI, informative and noninformative images). The workflow and example results of their proposed method are illustrated in Fig. 11. A total of 34,145 images were used for training, and 6000 images were used for testing (accuracy, 98.77%). Therefore, DL models can screen informatic frames as a preprocessing procedure. Then, other critical and computationally intensive models can perform only on the informatic frames, reducing the false-positive rate and leading to better real-time performance.

### Anatomical classification

**Fig. 12:**
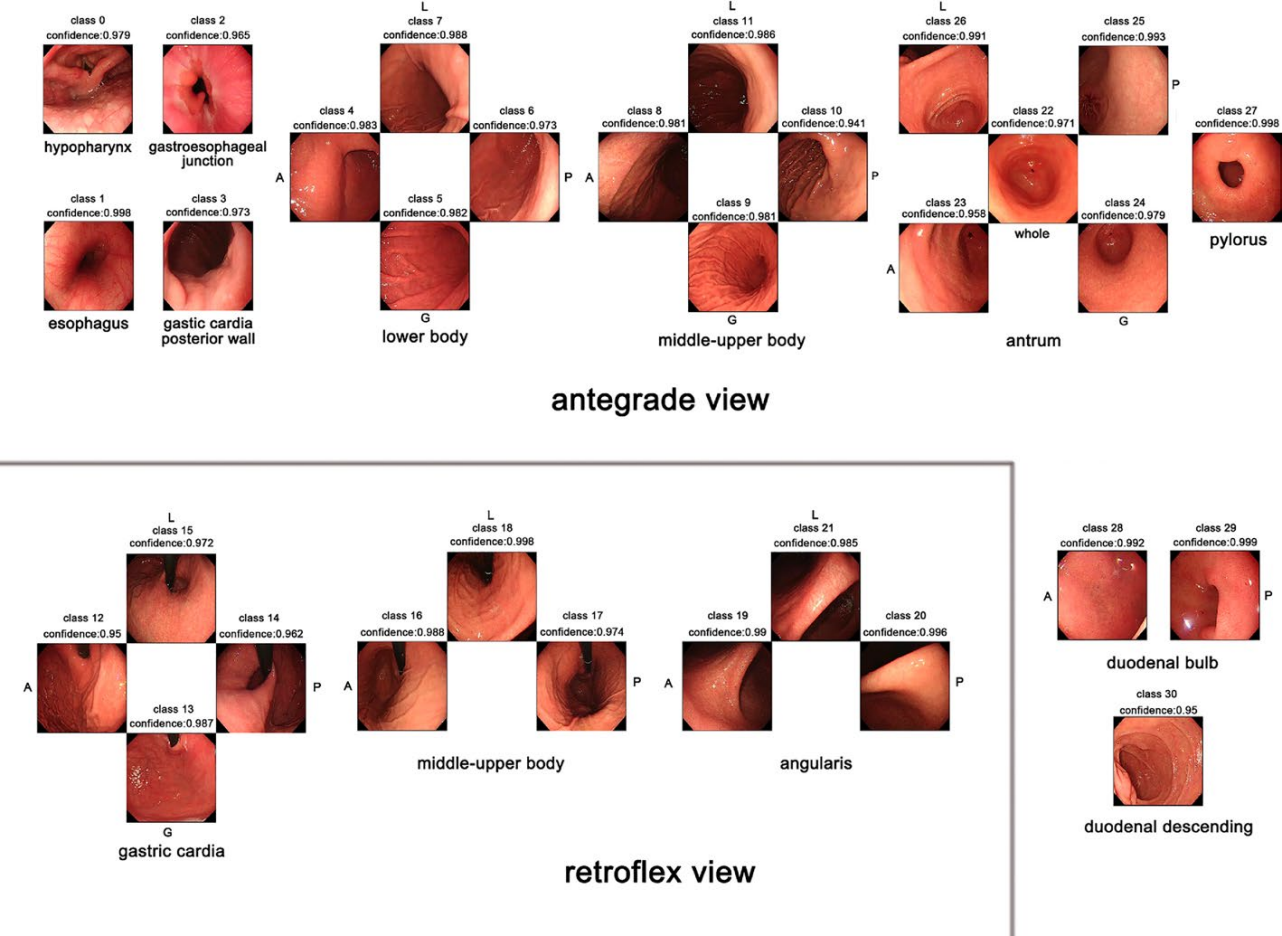
EGD images classified into 31 sites and representative images identified by IDEA. The images show 31 sites determined by IDEA and the prediction confidence. Class 0, hypopharynx; 1, oesophagus; 2, gastroesophageal junction; 3, gastric cardia P in the antegrade view; 4–7, lower body (A, G, P, L); 8–11, middle-upper body in the antegrade view (A, G, P, L); 12–15 gastric cardia in the retroflex view (A, G, P, L); 16–18, middle-upper body in retroflex view (A, P, L); 19–21, angularis (A, P, L); 22–26, antrum (whole, A, G, P, L); 27, pylorus; 28–29, duodenal bulb (A, P); 30, duodenal descending. A, anterior wall; G, greater curvature; P, posterior wall; L, lesser curvature. (Reproduced with permission from Ref. [2]. Copyright 2021 Elsevier Publishing)

While an endoscopist can capture all gastric cancer that appears under endoscopy, some lesions can be missed due to the wide, curved stomach lumen. Although guidelines for mapping the entire stomach exist, they are often not well followed. Therefore, it is important to develop a practicable and reliable algorithm to guide endoscopists to examine the stomach comprehensively. Takiyama et al. [23] constructed a CNN using GoogLeNet to classify the anatomical location of EGD images into the pharynx, oesophagus, upper stomach, middle stomach, lower stomach, and duodenum. An independent validation set of 17,081 EGD images was used to evaluate the model. The model showed an AUC of 1.00 for laryngeal and oesophageal images and 0.99 for stomach and duodenal images. Wu et al. [18] built a system (WISENSE, currently ENDOANGEL) to classify the anatomical locations of EGD images into 10 and 26 parts. The DCNN showed accuracies of 90% and 65.9% on real-time EGD videos with the two location classification tasks, respectively, comparable to the performance of experts (63.8%). Wu et al. also evaluated the system in a randomized controlled trial to ascertain whether the system can reduce the blind spot rate [12]. The blind spot rate was significantly lower on the WISENSE group than on the control group (5.86% vs. 22.46%). Additionally, a clinical trial was conducted to compare the performance of unsedated ultrathin transoral endoscopy (U-TOE), unsedated conventional oesophagogastroduodenoscopy (C-EGD), and sedated conventional oesophagogastroduodenoscopy (C-EGD) with or without the system. The blind spot rate was lowest on the sedated C-EGD, and the DL system reduced this rate to 3.42% [24]. It is more difficult to provide an accurate label using a single frame due to the refined division of anatomical locations and the variations in EGD performances among different individuals in practice. Therefore, using information from more adjacent frames is practicable. However, a CNN can only analyse frames independently. Li et al. [2] combined a DCNN (Inception-v3) and LSTM to develop a system (IDEA) to monitor blind spots during real-time EGD. A total of 170,297 images and 5779 endoscopic videos were used. The model could divide the EGD examination into 31 sites from the hypopharynx to the duodenum. Representative images identified by IDEA are shown in Fig. 12. In addition, an independent dataset of 3100 EGD images and 129 videos was used to evaluate its performance. The system showed a sensitivity, specificity, and accuracy of 97.18%, 99.91%, and 99.83%, respectively, for images and 96.29%, 93.32%, and 95.30%, respectively, for videos. Furthermore, IDEA using an NVIDIA GTX1080TI, a widely used affordable GPU, could process one image in 80 ms, thus meeting the real-time requirement. Zhang et al. [13] designed a CNN using SSD to detect 10 anatomical structures of the upper digestive tract in real time. The method showed a precision of 93.74%. The abovementioned studies are WLI-based. However, some image enhancement techniques, such as NBI, are commonly used in clinical practice. Igarashi et al. [25] developed an algorithm using AlexNet to classify EGD images into 14 precise anatomical categories under different image-capture conditions. The model showed an accuracy of 0.965 on the validation datasets with 36,072 images.

### Artefact detection

**Fig. 13:**
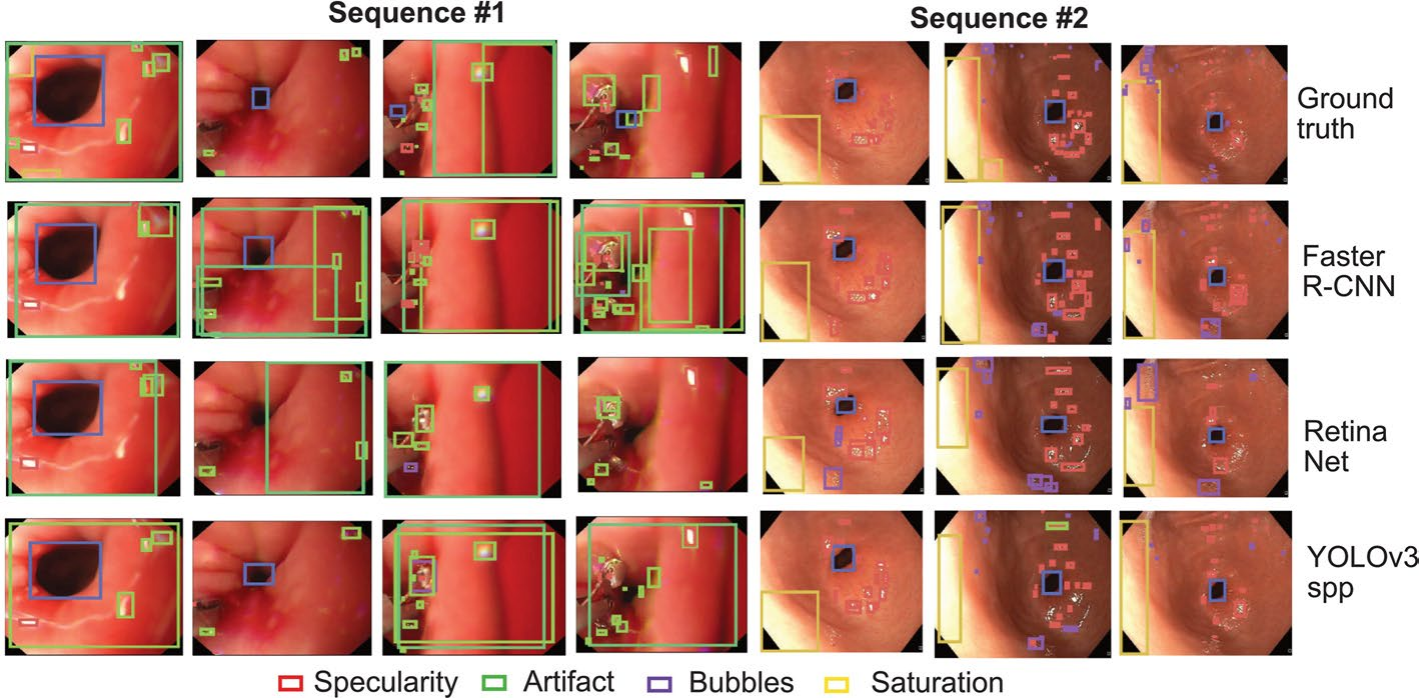
Artefact detection results on video sequence data with three object detection baselines. Bounding boxes for two different video sequences (each frame taken approximately after 10 frames). (Reproduced with permission from Ref. [7]. Copyright 2021 Elsevier Publishing)

Several artefacts, including motion blur, defocus, specularity reflection, over- and underexposure of image regions, and the presence of bubbles, fluids and artificial devices, corrupt over 60% of an endoscopy video frame, thus influencing the visual interpretation of the mucosal surface and significantly impeding the detection and quantitative analysis of lesions [95]. Therefore, it is important to identify and localize artefacts to restore video frame quality before developing other computer-assisted diagnosis algorithms. Figure 13 shows the results of three SOTA detection baselines on this challenge. Ali et al. [7] proposed a framework using deep learning to detect and classify six different primary artefacts and restore mildly corrupted frames. The method showed the highest mAP of 49.0 and the lowest computational time of 88 ms. The restoration model preserved an average of 68.7%, which is 25% more frames than that retained from the raw videos on 10 test videos. Ali et al. also held a computer vision challenge named Endoscopy Artefact Detection (EAD 2019 [96] and EAD 2020 [97]) and presented a comprehensive analysis of the submissions to EAD2019 [95] and EAD2020 [98].

### Depth estimation and 3D reconstruction of the stomach

**Fig. 14:**
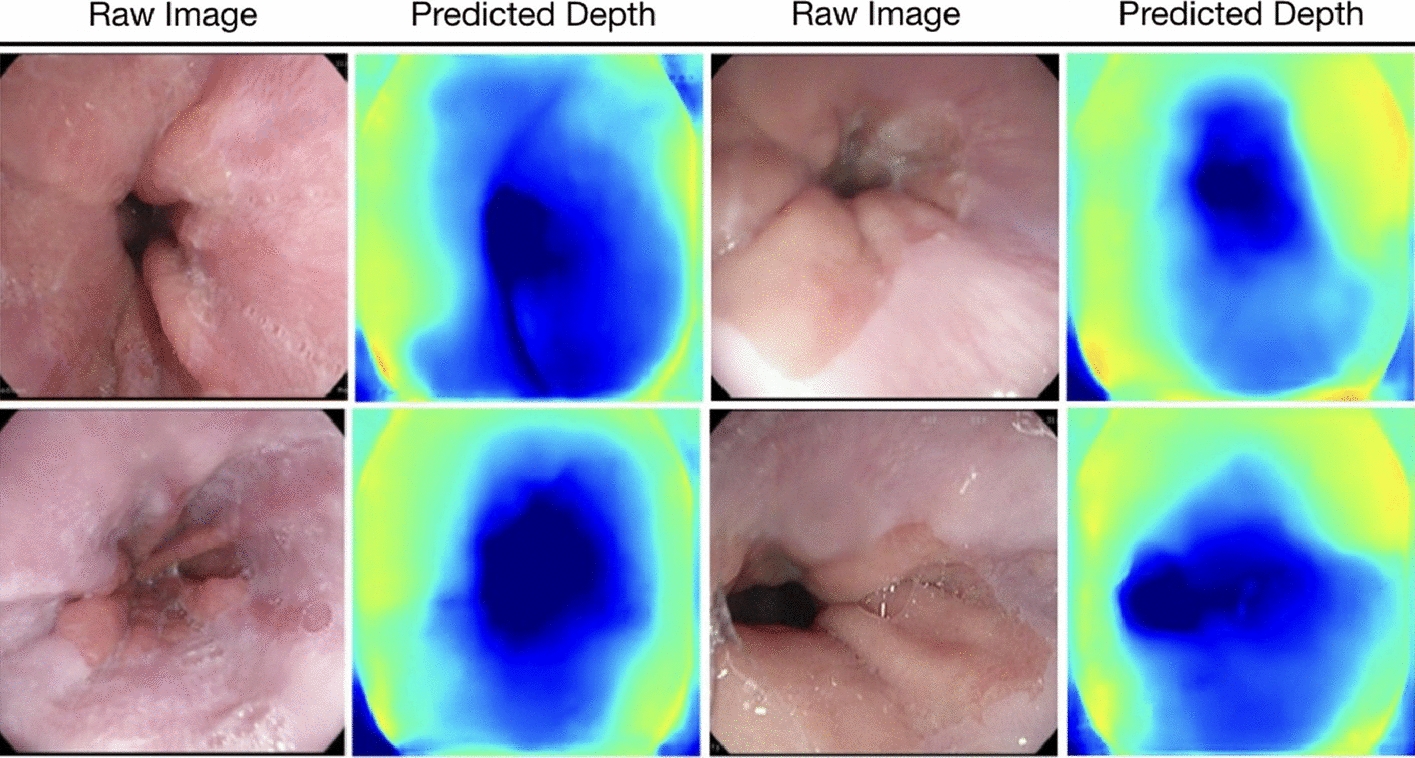
Depth estimation results for endoscopy data of the stomach. The model performed prediction on the Kvasir dataset for the stomach. Since there is no ground truth depth information available for this dataset, depth estimations are only provided with their corresponding raw images. (Reproduced with permission from Ref. [101]. Copyright 2021 Elsevier Publishing)

**Fig. 15:**
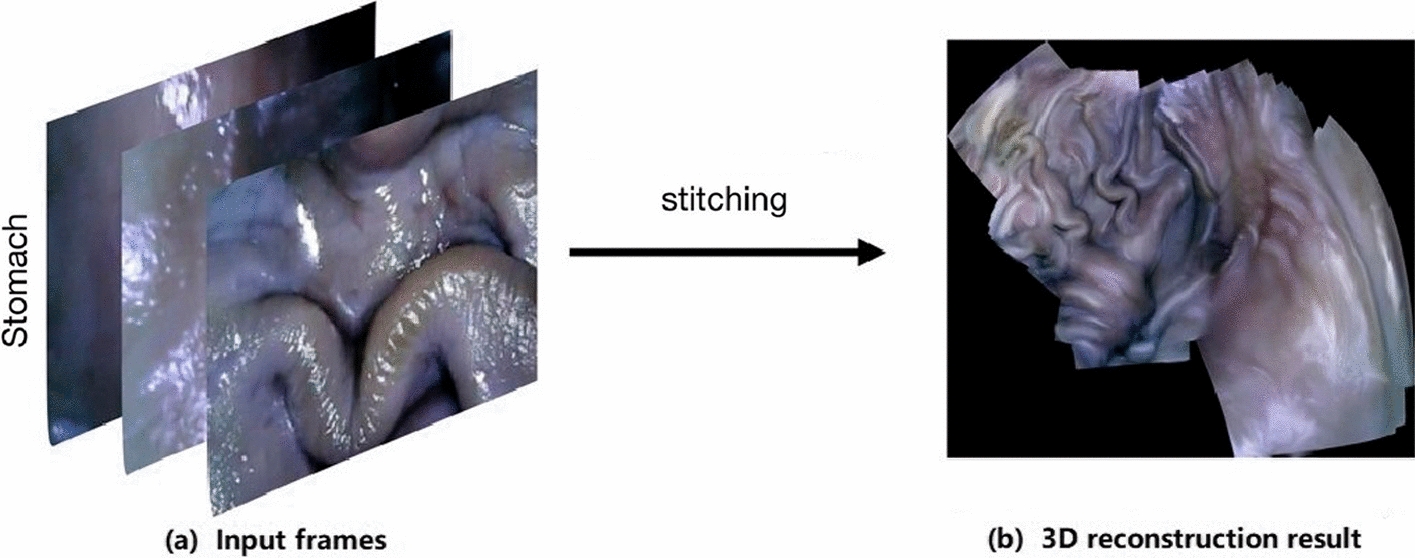
3D reconstruction results of the input frames from endoscopy data of the stomach. **a** Original frames from endoscopy data of the stomach. **b** Result of 3D reconstruction using these input frames. (Adapted with permission from Ref. [101]. Copyright 2021 Elsevier Publishing)

Conventional gastroscopy without 3D vision and proper depth perception significantly limits diagnostic examinations and therapy delivery. 3D surface reconstruction technology helps doctors better enhance scene perception on an augmented reality (AR) system, preventing surgical risks caused by low visibility and inexperience. In addition, 3D structural information can significantly improve diagnostic and surgical performance. Figures 14 and 15 explain the procedure of depth estimation and 3D reconstruction.

Recently, Widya et al. [6, 99, 100] used a chromoendoscopy video that spread indigo carmine (IC) dye on the stomach surface to reconstruct the entire 3D shape of the stomach with mucosal surface details via the structure from motion (SFM) method. The red channel data showed complete and comprehensive results. A network for image-to-image style translation from the no-IC image and the IC-sprayed image was trained using a generative adversarial network (GAN) to improve the previous work. Therefore, complete stomach 3D reconstruction can be performed without IC dying. Ozyoruk et al. [74] proposed an unsupervised monocular depth and pose estimation method that combines residual networks with spatial attention modules to focus on different and highly textured tissue regions. Moreover, a comprehensive endoscopic simultaneous localization and mapping (SLAM) dataset consisting of 3D point cloud data from ex vivo porcine gastrointestinal (GI) tract organs was built.

## Discussion

**Fig. 16:**
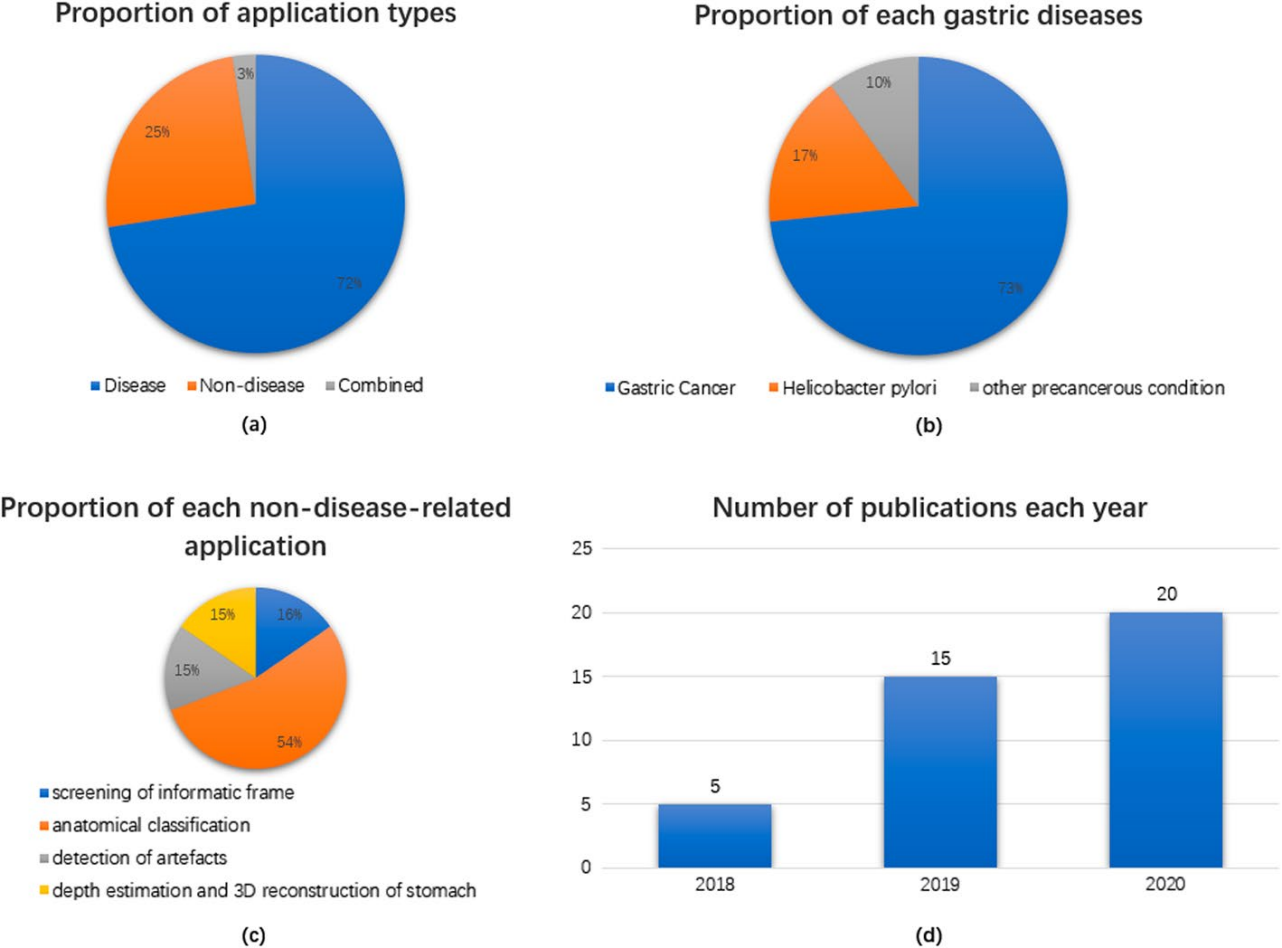
The statistical analysis of publications cited in this review. **a** The proportion of application types of deep learning to gastroscopy; **b** the percentage of each gastric disease in the disease-related application of deep learning; **c** the percentage of each nondisease-related application of deep learning; **d** the number of publications in this field each year

In recent years, increasing numbers of DL algorithms have been developed and successfully applied to natural image processing due to deep learning theory and the continuous improvement in hardware performance. Deep learning use in gastroscopy-assisted diagnosis is a new research hotspot. This review included 40 related papers. There is an increasing yearly trend based on the number of papers published. The articles included 29 applications related to diseases (see Table 1, mainly gastric cancer and Helicobacter pylori infection) and 10 not related to diseases (see Table 2, mainly monitoring the anatomical structure of the stomach to reduce blind spots). One paper also reported a system combining disease-related and nondisease-related applications to automatically detect EGC without blind spots. Figure 16 summarizes the publications cited in this review.

**Table 1:** Disease-related application of deep learning to gastroscopic image processing

Target disease	Main purpose	Reference	Imaging modality	DL task type	Dataset information	Network architecture	Result
GC	Detection of GC	Wang et al. [17]	WLI	Image classification	A total of 1350 images depicting cancer (highly suspicious) and 103,514 normal images Train:validation:test = 6:2:2	AlexNet GoogLeNet VGGNet	Sensitivity: 79.622% Specificity: 78.48% Misdiagnoses rate: 20.377% Misdiagnosis rate: 21.51%
		Hirasawa et al. [52]	WLI CE NBI	Object detection	Training dataset: 13,584 endoscopic images of gastric cancer Testing dataset: 2296 stomach images collected from 69 consecutive patients with 77 gastric cancer lesions	SSD	The CNN required 47 s to analyse 2296 test images The CNN correctly diagnosed 71 of 77 gastric cancer lesions with an overall sensitivity of 92.2% 161 noncancerous lesions were detected as gastric cancer, resulting in a positive predictive value of 30.6% 70 of the 71 lesions (98.6%) with a diameter of 6 mm or more as well as all invasive cancers were correctly detected
		Ishioka et al. [53]	WLI CE NBI	Object detection	Training dataset: 13,584 endoscopic images of gastric cancer Testing dataset: video images were collected from 68 endoscopic submucosal dissection procedures for early gastric cancer in 62 patients	SSD	The CNN correctly diagnosed 64 of 68 lesions (94.1%) Median time for lesion detection was 1 s (range: 0–44 s) after the lesions first appeared on the screen
		Ikenoyama et al. [55]	WLI CE NBI	Object detection	Training dataset: 13,584 endoscopic images from 2639 lesions of gastric cancer Testing dataset: 2940 images from 140 cases	SSD	The average diagnostic time for analysing 2940 test endoscopic images by the CNN and endoscopists were 45.5 ± 1.8 s and 173.0 ± 66.0 min, respectively The sensitivity, specificity, and positive and negative predictive values for the CNN were 58.4%, 87.3%, 26.0%, and 96.5%, respectively. These values for 67 endoscopists were 31.9%, 97.2%, 46.2%, and 94.9%, respectively The CNN had a significantly higher sensitivity than the endoscopists (by 26.5%)
		Luo et al. [72]	WLI	Semantic segmentation	A total of 1,036,496 endoscopy images from 84,424 individuals Train:validation:test = 8:1:1	DeepLabv3 +	The diagnostic accuracy in identifying upper gastrointestinal cancers was 0.955 in the internal validation set, 0.927 in the prospective set, and ranged from 0.915 to 0.977 in the five external validation sets The diagnostic sensitivity was similar to that of the expert endoscopist (0.942 vs. 0.945) and superior sensitivity compared with competent (0.858) and trainee (0.722) endoscopists The positive predictive value was 0.814 for the system, 0.932 for the expert endoscopist, 0.974 for the competent endoscopist, and 0.824 for the trainee endoscopist The negative predictive value was 0.978 for the system, 0.980 for the expert endoscopist, 0.951 for the competent endoscopist, and 0.904 for the trainee endoscopist
	Diagnosis of GC	Sakai et al. [16]	WLI	Image classification	Training dataset: 9587 cancer images and 9800 normal images Testing dataset: 4653 cancer images and 4997 normal images	GoogLeNet	Accuracy: 87.6% Sensitivity: 80.0% Specificity: 94.8%
		Cho et al. [26]	WLI	Image classification	Training dataset: 4205 images from 1057 patients Testing dataset: 812 images from 212 patients. An additional 200 images from 200 patients were collected and used for prospective validation	Inception-Resnet-v2	The weighted average accuracy of the model reached 84.6% for the five-category classification The mean area under the curve (AUC) of the model for differentiating gastric cancer and neoplasm was 0.877 and 0.927, respectively In prospective validation, the model showed lower performance compared with the endoscopist with the best performance (five-category accuracy 76.4% vs. 87.6%; cancer 76.0% vs. 97.5%; neoplasm 73.5% vs. 96.5%; P < 0.001). However, there was no significant difference between the model and the endoscopist with the worst performance in the differentiation of gastric cancer (accuracy 76.0% vs. 82.0%) and neoplasm (AUC 0.776 vs. 0.865)
		Lee et al. [27]	WLI	Image classification	Training dataset: 200 Ulcer images, 337 cancer images, 180 normal images Testing dataset: 20 ulcer images, 30 cancer images, 20 normal images	ResNet-50 VGGNet-16 Inception v4	The AUCs were 0.95, 0.97, and 0.85 for the three classifiers. ResNet-50 showed the highest level of performance The cases involving normal, i.e., normal vs. ulcer and normal vs. cancer resulted in accuracies above 90%. The case of ulcer vs. cancer classification resulted in a lower accuracy of 77.1%,
		Li et al. [34]	ME-NBI	Image classification	Training dataset: A total of 386 images of noncancerous lesions and 1702 images of early gastric cancer Testing dataset: A total of 341 endoscopic images (171 noncancerous lesions and 170 early gastric cancer)	Inception-v3	The sensitivity, specificity, and accuracy of the CNN system in the diagnosis of early gastric cancer were 91.18%, 90.64%, and 90.91%, respectively No significant difference in the specificity and accuracy of diagnosis between the CNN and experts. However, the diagnostic sensitivity of the CNN was significantly higher than that of the experts The diagnostic sensitivity, specificity and accuracy of the CNN were significantly higher than those of the nonexperts
		Horiuchi et al. [31]	ME-NBI	Image classification	Training dataset: 1492 EGC and 1078 gastritis images Testing dataset: 151 EGC and 107 gastritis images	GoogLeNet	Accuracy: 85.3% Sensitivity: 95.4% Specificity: 71.0% PPV: 82.3% NPV: 91.7% The overall test speed was 51.83 images/s (0.02 s/image)
		Horiuchi et al. [32]	ME-NBI	Image classification	Training dataset: 1492 cancerous and 1078 noncancerous images obtained using ME-NBI Testing dataset: 174 videos (87 cancerous and 87 noncancerous videos) Comparisons were made between the system and 11 experts who were skilled in diagnosing EGC using ME-NBI with clinical experience of more than 1 year	GoogLeNet	AUC: 0.8684 Accuracy: 85.1% Sensitivity: 87.4% Specificity: 82.8% PPV: 83.5% NPV: 86.7% The CAD system was significantly more accurate than two experts, significantly less accurate than one expert, and not significantly different from the remaining eight experts
		Hu et al. [33]	ME-NBI	Image classification	A total of 1777 ME-NBI images from 295 cases were collected from 3 centres Training cohort (TC, n = 170) Internal test cohort (ITC, n = 73) External test cohort (ETC, n = 52) compared the model with eight endoscopists with varying experience	VGG-19	AUC: 0.808 in the ITC and 0.813 in the ETC Similar predictive performance to the senior endoscopists (accuracy: 0.770 vs. 0.755; sensitivity: 0.792 vs. 0.767; specificity: 0.745 vs. 0.742) better than the junior endoscopists (accuracy: 0.770 vs. 0.728) After referring to the results of the system, the average diagnostic ability of the endoscopists was significantly improved in terms of accuracy, sensitivity, PPV, and NPV
		Liu et al. [36]	ME-NBI	Image classification	A total of 3871 ME-NBI images including 1130 CGT, 1114 LGN and 1627 EGC tenfold cross-validation	ResNet-50 VGG-16 Inception-v3 InceptionResNetv2	ResNet-50 is the best among the four networks Accuracy: 0.96 f1-scores: 0.92, 0.91 and 0.99 for classifying ME-NBI images into CGT, LGN and EGC, respectively
		Ueyama et al. [38]	ME-NBI	Image classification	Training dataset: 5574 ME‐NBI images (3797 EGCs, 1777 non‐cancerous mucosa and lesions) Testing dataset: 2300 ME‐NBI images (1430 EGCs, 870 non‐cancerous mucosa and lesions)	ResNet-50	The AI‐assisted CNN‐CAD system required 60 s to analyse 2300 test images Accuracy: 98.7% Sensitivity: 98% Specificity: 100% Positive predictive value: 100% Negative predictive value: 96.8% All misdiagnosed images of EGCs were of low‐quality or of superficially depressed and intestinal‐type intramucosal cancers that were difficult to distinguish from gastritis, even by experienced endoscopists
		Zhang et al. [39]	WLI	Image classification Semantic segmentation	Training dataset: 21,217 gastroscopic images of peptic ulcer (PU), early gastric cancer (EGC), high‐grade intraepithelial neoplasia (HGIN), advanced gastric cancer (AGC), gastric submucosal tumours (SMTs), and normal gastric mucosa without lesions Testing dataset: 1091 images The CNN diagnosis was compared with those of 10 endoscopists with over 8 years of experience in endoscopic diagnosis	ResNet34 DeepLabv3	The diagnostic specificity and PPV of the CNN were higher than that of the endoscopists for the EGC and HGIN images (specificity: 91.2% vs. 86.7%; PPV: 55.4% vs. 41.7%) The diagnostic accuracy of the CNN was close to those of the endoscopists for the lesion‐free, EGC and HGIN, PU, AGC, and SMTs images The CNN had an image recognition time of 42 s for all the test set images
	Determining the invasion depth of GC	Zhu et al. [29]	WLI	Image classification	Training dataset: 790 images Testing dataset: 203 images	ResNet-50	At a threshold value of 0.5, the sensitivity was 76.47%, the specificity was 95.56%, the AUC was 0.94, the overall accuracy was 89.16%, the positive predictive value was 89.66%, and the negative predictive value was 88.97% The CNN–CAD system achieved significantly higher accuracy (by 17.25%) and specificity (by 32.21%) than human endoscopists
		Cho et al. [30]	WLI	Image classification	Internal dataset: a total of 2899 images Train:validation:test = 8:1:1 External dataset: 206 images for testing	DenseNet-161	In the internal test, the mean area under the curve discriminating submucosal invasion was 0.887 In the external test, the mean area under the curve reached 0.887 Clinical simulation showed that 6.7% of patients who underwent gastrectomy in the external test were accurately qualified by the established algorithm for potential endoscopic resection, avoiding unnecessary operation
	Delineating the margin of GC	An et al. [73]	WLI CE ME-NBI	Semantic segmentation	Training dataset: WLI: 343 images from 260 patients CE: 546 images from 67 patients Testing dataset: WLI: 321 images from 218 patients CE: 34 images from 14 patients	UNet + +	The system had an accuracy of 85.7% on the CE images and 88.9% on the WLE images under an overlap ratio threshold of 0.60 in comparison with the manual markers labelled by the experts On the ESD videos, the resection margins predicted by the system covered all areas of high-grade intraepithelial neoplasia and cancers The minimum distance between the margins predicted by the system and the histological cancer boundary was 3.44 ± 1.45 mm which outperformed the resection margin based on ME-NBI
	Detection of GC Anatomical classification	Wu et al. [18]	NBI BLI WLI	Image classification	Training dataset: 3170 gastric cancer and 5981 benign images for detect GC; 24,549 images from different parts of stomach for monitor blind spots Testing dataset: 100 gastric cancer and 100 benign images for detect GC; 170 images for monitor blind spots	VGG-16 ResNet-50	The DCNN identified EGC from nonmalignancy with an accuracy of 92.5%, a sensitivity of 94.0%, a specificity of 91.0%, a positive predictive value of 91.3%, and a negative predictive value of 93.8% The DCNN classified gastric locations into 10 or 26 parts with an accuracy of 90% or 65.9%
	Detection of GC Determining the invasion depth of GC	Yoon et al. [19]	WLI	Image classification	A total of 11,539 images (896 T1a-EGC, 809 T1b-EGC, and 9834 non-EGC) Train:validation:test = 6:2:2	VGG-16	AUC for EGC detection: 0.981 AUC for depth prediction: 0.851
	Detection of GC Delineating the margin of GC	Shibata et al. [82]	WLI	Image classification Semantic segmentation	A total of 1208 healthy and 533 cancer images fivefold cross-validation	Mask R-CNN	For the detection task: the sensitivity and false positives (FPs) per image were 96.0% and 0.10 FP/image, respectively For segmentation task: the average Dice index was 71%
	Classifying the type of GC Delineating the margin of GC	Ling et al. [35]	ME-NBI	Image classification	For CNN1 to identify EGC differentiation status Training dataset: 2217 images from 145 EGC patient Testing dataset: 1870 images from 139 EGC patients The performance of CNN1 was then compared with that of experts using 882 images from 58 EGC patients For CNN2 to delineate the EGC margins Training dataset: 928 images from 132 EGC patients Testing dataset: 742 images from 87 EGC patients	VGG-16 and ResNet-50 UNet + +	The system predicted the differentiation status of EGCs with an accuracy of 83.3% in the testing dataset In the man – machine contest, CNN1 performed significantly better than the five experts (86.2% vs. 69.7%) The system delineated EGC margins with an accuracy of 82.7% in differentiated EGC and 88.1% in undifferentiated EGC under an overlap ratio of 0.80 In unprocessed EGC videos, the system achieved real-time diagnosis of EGC differentiation status and EGC margin delineation in ME-NBI endoscopy
HP	Detection of HP	Itoh et al. [14]	WLI	Image classification	179 upper gastrointestinal endoscopy images obtained from 139 patients (65 were HP-positive and 74 were HP-negative) Training dataset: 149 were used as training images, The 149 training images were subjected to data augmentation, which yielded 596 images Testing dataset: the remaining 30 (15 from HP-negative patients and 15 from HP-positive patients) were set aside to be used as test images	GoogLeNet	Sensitivity: 86.7% Specificity: 86.7% AUC: 0.956
		Nakashima et al. [15]	WLI BLI LCI	Image classification	Training dataset: For per group (WLI BLI LCI): 486 images (rotated 90, 180, and 270 degrees) in addition to the original 162, for a total of 648 Testing dataset: For per group (WLI BLI LCI): 60 images	GoogLeNet	AUC for WLI: 0.66 AUC for BLI: 0.96 AUC for LCI: 0.95
		Zheng et al [20]	WLI	Image classification	Training dataset: 11,729 gastric images Testing dataset: 3755 gastric images	ResNet-50	The AUC for a single gastric image was 0.93 with sensitivity, specificity, and accuracy of 81.4%, 90.1%, and 84.5%, respectively, using an optimal cut-off value of 0.3 The AUC for multiple gastric images per patient was 0.97 with sensitivity, specificity, and accuracy of 91.6%, 98.6%, and 93.8%, respectively, using an optimal cut-off value of 0.4
	Diagnosis of HP	Nakashima et al. [37]	LCI WLI	Image classification	Training dataset: 6639 WLI images and 6248 LCI images from 395 subjects Testing dataset: Videos of 120 subjects	A 22-layer skip-connection architecture	For the LCI-CAD system: Accuracy: 84.2% for uninfected, 82.5% for currently infected, and 79.2% for post-eradication status For the WLI-CAD system: Accuracy: 75.0% for uninfected, 77.5% for currently infected, and 74.2% for post-eradication status The LCI-CAD system demonstrated significantly superior diagnostic accuracy to that of the WLI-CAD system and comparable diagnostic accuracy to that of experienced endoscopists
		Shichijo et al. [28]	WLI	Object detection	Training dataset: 98,564 endoscopic images from 5236 patients (742 H. pylori-positive, 3649 -negative, and 845 -eradicated) Testing dataset: 23,699 images from 847 patients; 70 positive, 493 negative, and 284 eradicated	GoogLeNet	80% (465/582) of negative diagnoses were accurate, 84% (147/174) eradicated, and 48% (44/91) positive The time needed to diagnose 23,699 images was 261 s
GP	Detection of GP	Zhang et al. [54]	WLI	Image classification	Training dataset: 708 images Testing dataset: 50 images	SSD	The model can realize real-time polyp detection with 50 frames per second (FPS) The model can achieve the mean average precision (mAP) of 90.4% The model has excellent performance in improving polyp detection recalls over 10%, especially in small polyp detection
GIM	Diagnosis of GIM	Yan et al. [22]	NBI ME-NBI	Image classification	Training dataset: 1880 endoscopic images (1048 GIM and 832 non-GIM) from 336 patients Testing dataset: 477 pathologically confirmed images (242 GIM and 235 non-GIM) from 80 patients	EfficientNetB4	AUC: 0.928 Sensitivity: 91.9% Specificity: 86.0% Accuracy: 88.8%
CAG	Diagnosis of CAG	Zhang et al. [21]	White-light i-Scan	Image classification	A total of 5470 images of the gastric antrum of 1699 patients (3042 images depicted atrophic gastritis and 2428 did not) fivefold cross-validation The diagnoses of the deep learning model were compared with those of three experts	DenseNet121	Accuracy: 0.942 Sensitivity: 0.945 Specificity: 0.940 The detection rates of mild, moderate, and severe atrophic gastritis were 93%, 95%, and 99%, respectively The diagnostic performance of the CNN model was higher than that of the experts

WLI, white-light imaging; CE, chromoendoscopy; NBI, narrow-band imaging; GC, gastric cancer; SSD, single-shot multibox detection; CNN, convolutional neural network; HP, Helicobacter pylori; AUC, area under curve; BLI, blue-light imaging; LCI, linked colour imaging; DCNN, deep convolutional neural network; EGC, early gastric cancer; FPS, frame per second; mAP, mean average precision; GP, gastric polyp; CAD, computer-aided diagnosis; WLE, white-light endoscopy; ESD, endoscopic submucosal dissection; ME, magnifying endoscope; PPV, positive predictive value; NPV, negative predictive value; CGT, chronic gastritis; LGN, low-grade neoplasia; AI, artificial intelligence; GIM, gastric intestinal metaplasia; PU, peptic ulcer; HGIN, high-grade intraepithelial neoplasia; AGC, advanced gastric cancer; SMTs, submucosal tumours; CAG, chronic atrophic gastritis

**Table 2:** Non-disease-related application of deep learning in gastroscopic image processing

Main purpose	Reference	Imaging modality	DL task type	Dataset information	Network Architecture	Result
Anatomical classification	Takiyama et al. [23]	WLI	Image classification	Training dataset: 27,335 EGD images categorized into four major anatomical locations (larynx, oesophagus, stomach and duodenum) and three subsequent subclassifications for stomach images (upper, middle, and lower regions) Testing dataset: 17,081 EGD images	GoogLeNet	AUCs of 1.00 for larynx and oesophagus images, and 0.99 for stomach and duodenum images AUCs of 0.99 for the upper, middle, and lower stomach within the stomach
	Chen et al. [24]	WLI	Image classification	437 patients were randomized to unsedated U-TOE, unsedated C-EGD, or sedated C-EGD, and each group was divided into two subgroups: with or without the assistance of a DL system to monitor blind spots during EGD	VGG-16	The blind spot rate with DL-assisted sedated C-EGD was significantly lower than that of unsedated U-TOE and unsedated C-EGD (3.42% vs. 21.77% vs. 31.23%) The blind spot rate of the DL subgroup was lower than that of the control subgroup in all 3 groups (sedated C-EGD: 3.42% vs. 22.46%; unsedated U-TOE: 21.77% vs. 29.92%; unsedated C-EGD: 31.23% vs. 42.46%)
	Igarashi et al. [25]	WLI NBI CE	Image classification	A total of 85,246 raw upper GI endoscopic images from 441 patients with gastric cancer Training dataset: 49,174 images Testing dataset: 36,072 images	AlexNet	Accuracy: 0.965
	Li et al. [2]	WLI	Image classification	Training dataset: 170,297 images and 5779 endoscopic videos Testing dataset: 3100 EGD images and 129 videos	Inception-v3 + LSTM	For images: the sensitivity, specificity, and accuracy of DCNN were determined as 97.18%,99.91%, and 99.83%, respectively For videos: the sensitivity, specificity, and accuracy of DCNN were 96.29%,93.32%, and 95.30%, respectively The DCNN was able to process one image in 80 ms using an NVIDIA GTX1080TI GPU, which means the frame rate performance of this model was 12.5 fps, thus meeting the real-time requirement
Detection of artefacts	Ali et al. [102]	WLI NBI	Object detection	A total of 1290 endoscopic images from seven unique patient videos, of which 1229 images are WLI, and 61 images are NBI Training dataset: 90% of total, 1161 images Testing dataset: 10% of total, 129 images	YOLO v3-spp	mAP of 45.7 at IoU thresholds 0.25 Overall mAP of 30.63 Detection speed of 88 ms per image
	Zhang et al. [56]	WLI NBI	Object detection	Training dataset: 2322 images Validation dataset: 291 images Testing dataset: 195 images	Cascade RCNN	Score_d (0.6 * Map + 0.4 * IoU): 0.3429
Depth estimation and 3D reconstruction	Widya et al. [6]	WLI CE	Semantic segmentation	Training dataset: 7978 no-IC images and 7453 IC-sprayed images Testing dataset: 7 subjects	CycleGAN	The generated VIC images from cycleGAN achieve better results on all subjects compared to the baseline no-IC green-channel images Using the VIC images for SfM significantly improves the number of reconstructed images All reconstruction results using the VIC images achieve more than 95% of reconstructed images The triangulated 3D points also demonstrate significant improvement
	Ozyoruk et al. [74]	N/A	Semantic segmentation	A total of 42,700 images from ex vivo porcine Testing dataset: 1548 stomach frames from ex vivo porcine	spatial attention based ResNet	The RMSE values of stomach are 0.41 cm for depth estimation using Endo-SfMLearner
Screening of informatic frames Anatomical classification	Wu et al. [12]	WLI	Image classification	Training dataset: 12,220 in vitro, 25,222 in vivo and 16,760 unqualified EGD images of over 3000 patients for training the network to identify whether a scope was in or outside the body (DCNN1). 34,513 qualified EGD images labelled into 26 different sites for training the network of classifying gastric sites (DCNN2) Testing dataset: A total of 107 stored EGD videos A total of 324 patients were recruited and randomized. 153 and 150 patients were analysed in the system-assisted group and control group, respectively	VGG-16	The system monitored blind spots with an accuracy of 90.40% in EGD videos Blind spot rate was lower in system-assisted group compared with the control group (5.86% vs. 22.46%),
	Xu et al. [13]	WLI NBI	Image classification Object detection	Training dataset: For the classification task: 34,145 images For the detection task: 47,623 images Testing dataset: For the classification task: 6000 images For the detection task: 12,600 images	SSD	93.74% mean average precision (Map) for the detection task 98.77% accuracy for the classification task
Detection of GC Anatomical classification	Wu et al. [18]	NBI BLI WLI	Image classification	Training dataset: 3170 gastric cancer and 5981 benign images for detect GC 24,549 images from different parts of stomach for monitor blind spots Testing dataset: 100 gastric cancer and 100 benign images for detecting GC, 170 images for monitor blind spots	VGG-16 ResNet-50	The DCNN identified EGC from nonmalignancy with an accuracy of 92.5%, a sensitivity of 94.0%, a specificity of 91.0%, a positive predictive value of 91.3%, and a negative predictive value of 93.8% The DCNN classified gastric locations into 10 or 26 parts with an accuracy of 90% or 65.9%

WLI, white-light imaging; EGD, oesophagogastroduodenoscopy; AUC, area under curve; DCNN, deep convolutional neural network; NBI, narrow-band imaging; BLI, blue-light imaging; GC, gastric cancer; EGC, early gastric cancer; mAP, mean average precision; IoU, intersection over union; U-TOE, ultrathin transoral endoscopy; C-EGD, conventional oesophagogastroduodenoscopy; DL, deep learning; CE, chromoendoscopy; GI, gastrointestinal; LSTM, long short-term memory networks; IC, indigo carmine; VIC, virtual indigo carmine; SfM, structure from motion; RMSE, root mean square error

To date, some systems using DL in gastroscopy have worked under real-time video conditions and achieved technical indicators comparable to expert endoscopists in both disease-related and nondisease-related applications. However, some key issues should be addressed before clinical use. First, most studies used retrospective datasets based on high-quality static images. When these models are used in real-time video analysis, performance tends to be poor due to the relatively poor quality of the video frames. Therefore, more prospective studies using video images are needed. Additionally, the current research used a small dataset due to the privacy of patients and the high cost of labelling the images, and unignorable selection bias existed. Although the performance in each study was high, the algorithms cannot be compared because there was no unified benchmark using the same dataset, such as ImageNet and MS COCO for natural image analysis. Large-scale open-access databases, such as the SUN database for a colonoscopy, should be used [103]. Furthermore, the clinical value can only be known by deploying the system in hospitals, which requires the approval of relevant regulatory authorities. Although some regulatory-approved DL systems are available for colonoscopy [104–107], there is no such system for gastroscopy. Therefore, regulatory considerations for deep learning technologies in gastroscopy should be given more attention by major regulatory authorities [Food and Drug Administration (FDA, US); Pharmaceuticals and Medical Devices Agency (PMDA, Japan); National Medical Products Administration (NMPA, China); European Conformity (CE, Europe)] [108].

### Future perspective for disease-related DL application to gastroscopy

It is necessary to develop a system that can detect key diseases in the stomach at the same time to make the system comprehensive in a pathological sense. The systems in this research are only sensitive to one disease, such as GC or HP, and are exclusive. This is not effective in clinical practice and can easily hinder an endoscopist’s examination. For instance, an HP detection system is not sensitive to GC lesions. The system could not give a reminder to the endoscopist when a GC lesion appeared on the screen, thus leading to missing data.

Furthermore, the system should achieve higher performance on some disease subtypes that endoscopists easily miss, such as lesions with a specific pathological status, a specific location, or a specific size; otherwise, if high technical metrics are achieved only on some lesions that are rarely ignored by physicians, then the system will have no great clinical significance.

In addition, there is a 2- to 3-year gap for deep learning technology application in gastroscopy compared to most cutting-edge research. Most state-of-the-art algorithms in deep learning have not been applied to screen diseases under gastroscopy. For instance, a 3D object detection algorithm can significantly improve the detection performance of flat lesions compared with a 2D object detection algorithm because it provides in-depth information. Some algorithms sensitive to small objects with only a few image pixels [109], camouflaged objects that are difficult to distinguish from a background [110], and few-shot or even zero-shot objects rarely appearing in small datasets [111] have been developed and applied to natural images and are important in gastroscopic lesion detection. However, it has not been applied in gastroscopic image analysis. In this research field, researchers often directly use algorithms that have achieved good results on natural images and perform transfer learning to obtain their models without making any changes to the network structure based on prior knowledge to make it more suitable for endoscopy image analysis. However, there are significant differences between endoscopic images and natural images in colour or texture. Therefore, doctors need to cooperate with DL algorithm engineers.

### Future perspective for nondisease-related DL application to gastroscopy

Deep learning for nondisease-related applications enables disease-related applications to achieve better performance.

First, a nondisease-related DL model should make a disease-related model effectively detect and diagnose lesions to suit the real-time requirement. Therefore, more lightweight models with fewer parameters and inference calculations should be adopted. In addition, it will screen the frames with no information (motion blur, defocus.) and those with unsuitable imaging modalities (WLI, NBI, ME.). A relatively time-consuming disease-related model should only analyse the informatic frames after screening. In addition, the most appropriate endoscopy imaging modality based on the task settings should be clarified.

For the gastroscopy coverage rate, a nondisease-related DL model should enable a disease-related model to comprehensively inspect the stomach, covering the entire mucosal surface of the stomach without visual obstruction. Combining deep neural networks such as CNNs, RNNs and GANs should be explored. Currently, researchers perform anatomical classification of video frames to ensure gastroscopy comprehensiveness. However, the performance of this method decreases with detailed anatomical classification (classification of the stomach from 10 to 26 regions). Combining a CNN and RNN, which is more powerful in serialized video data processing, significantly increases the performance of the DL model up to 31 regions for the classification task.

Furthermore, some significant additional functions for gastroscopy can be realized using DL technology to solve clinical limitations. For instance, a monocular visual odometer with deep learning can be used to accurately measure lesion size, which is important for the diagnosis, treatment, and prognosis of a lesion. However, endoscopists currently estimate lesion size by comparing it with a reference object such as biopsy forceps, which has unignorable errors. In some nonmedical fields, such as automatic driving, visual measurement technology based on deep learning is a hot research direction. In the field of endoscopy, the newest research [112] showed clear boundaries in estimated depth by resampling pixels around occlusion boundaries. One obstacle was that the texture of tissue is patient-specific when first used for depth reconstruction of colonoscopy [113]. While monocular methods are most effective without other attachments, the images obtained are the same for the motion of the monocular camera, zooming trail, and scene in the same multiple (since the epipolar constraint is equal to 0 [131]). Therefore, the object scale cannot be obtained via monocular-based methods. Solving the problem of the lack of measurement scale of a monocular endoscope will become an important challenge.

### Promising techniques and approaches of DL for gastroscopy

Currently, several cutting-edge DL technologies have attracted widespread attention in the field of natural image processing. They have been proven to bring great improvements in medical image processing, such as MRIs, CTs, and X-rays, but have never been applied to gastroscopic image processing.

In terms of network architecture, a transformer based on an attention mechanism can extract more global features of an image than a CNN. Representative approaches such as ViT [114], DETR [115], SETR [116], and Swin-T [117] have obtained better results than a CNN for the classification, detection, and segmentation of natural images. In the field of medical image processing, some recent research, such as MedT [118], Swin-UNet [119], and SpecTr [120], have achieved SOTA performance on brain ultrasound image segmentation, gland microscope image segmentation, and multiorgan CT image segmentation.

In addition, network architecture search (NAS) is another direction of network architecture development. There is a large difference in semantics between medical images and natural images. Therefore, a network structure that achieves good results on natural images is not necessarily suitable for medical images. Redesigning a network structure for medical images requires a wealth of expertise. A NAS algorithm can reduce the need for prior knowledge and automatically search for an optimal network structure. Some well-known works in the NAS field, such as the DARTS series [121–123] and ProxylessNAS [124], have achieved surprising performance in natural image analysis. Recently, some studies on medical image processing have introduced NAS. For example, NAS-UNet [125], AutoDeepLab [126], MS-NAS [127], and BiX-NAS [128] have achieved SOTA performance on medical image segmentation.

For the training paradigm, self-supervised learning is a promising technology. Due to the complexity of medical images, doctors with professional knowledge are required to annotate images. This results in the scale of labelled medical image datasets always being small. In contrast, unlabelled raw medical images are relatively easy to obtain. To solve this problem, self-supervised learning methods such as the MoCo series [129–131], SimCLR series [132, 133], and BYOL [134] are considered, which can be trained using unlabelled data and have achieved comparable performance to supervised learning methods on natural image datasets. Studies based on these approaches, such as MoCo-CXR [135] and MedAug [136], have recently been applied to detect abnormalities in chest X-ray images.

Regarding the optimization procedure, currently applied optimizers usually utilize the gradient descent of the loss function to find an optimal solution. However, these optimization technologies are susceptible to the local optimal trap. Recently, some meta-heuristic algorithms, such as the Aquila Optimizer (AO) [137], Reptile Search Algorithm (RSA) [138] and Arithmetic Optimization Algorithm (AOA) [139], have been employed to solve a variety of complicated optimization problems. These optimization algorithms are able to perform a global search in the available search space of a problem to ensure that the final solution is close to the global optimum, which demonstrates the potential to improve the optimization process of developing DL models for gastroscopy.

## Conclusion

Based on the findings mentioned above, we suggest that a DL-based assisted system for real-time gastroscopy to provide on-site support should be developed in a manner combining deep learning applications in disease-related and nondisease-related situations. Four development trends of deep learning in gastroscopy can be observed from the literature cited in this review: (1) real-time performance is improved; (2) coverage comprehensiveness (in both a spatial sense and pathological sense) is achieved; (3) detection sensitivity is enhanced; and (4) diagnosis accuracy is increased. However, there is still a gap before these systems can be applied to clinical practice. In the future, it is important to test the complete system using clinical indicators after validating a single function at the algorithm level using technical metrics such as sensitivity, specificity, PPV, and NPV, which are easily affected by the distribution of the test dataset. Another potential research direction is to conduct multicentre randomized controlled trials to test whether the system can improve the performance of endoscopists in an actual clinical environment, reduce the blind spot rate, increase the detection rate, and reduce the incidence of fatal, high-burden, and poor prognosis diseases such as advanced cancers. Furthermore, the exploration of more cutting-edge DL algorithms and their potential applications that are beneficial to gastroscopy can be future work for the research community. In conclusion, deep learning has the potential to improve the efficiency and quality of gastroscopy soon. However, endoscopists should first understand what DL can do and how to use it.

## Data Availability

Not applicable.

## Abbreviations

NBI: Narrow band imaging
ME: Magnifying endoscope
AFI: Autofluorescence imaging
DL: Deep learning
SOTA: State-of-the-art
ML: Machine learning
CNN: Convolutional neural network
EGD: Esophagogastroduodenoscopy
RNN: Recurrent neural network
GAN: Generative adversarial network
HP: Helicobacter pylori
WLI: White-light imaging
AUC: Area under curve
BLI: Blue-light imaging
LCI: Linked colour imaging
CAD: Computer-aided diagnosis
GC: Gastric cancer
SSD: Single shot multibox detection
EGC: Early gastric cancer
ESD: Endoscopic submucosal dissection
GI: Gastrointestinal
DCNN: Deep convolutional neural network
PPV: Positive predictive value
NPV: Negative predictive value
AGC: Advanced gastric cancer
HGD: High-grade dysplasia
LGD: Low-grade dysplasia
PU: Peptic ulcer
HGIN: High-grade intraepithelial neoplasia
SMTs: Submucosal tumours
CGT: Chronic gastritis
LGN: Low-grade neoplasia
CE: Chromoendoscopy
WLE: White-light endoscopy
mAP: Mean average precision
GIM: Gastric intestinal metaplasia
U-TOE: Ultrathin transoral endoscopy
C-EGD: Conventional esophagogastroduodenoscopy
LSTM: Long Short-Term Memory networks
SFM: Structure from motion
IC: Indigo carmine
SLAM: Simultaneous localization and mapping
